# Comparative Genomics and Phylogenomics of Hemotrophic Mycoplasmas

**DOI:** 10.1371/journal.pone.0091445

**Published:** 2014-03-18

**Authors:** Ana M. S. Guimaraes, Andrea P. Santos, Naíla C. do Nascimento, Jorge Timenetsky, Joanne B. Messick

**Affiliations:** 1 Department of Comparative Pathobiology, School of Veterinary Medicine, Purdue University, West Lafayette, Indiana, United States of America; 2 Coordenação de Aperfeiçoamento de Pessoal de Ensino Superior (CAPES)-Fulbright Program, Ministério da Educação, Brasília, Distrito Federal, Brazil; 3 Department of Microbiology, Instituto de Ciências Biomédicas, Universidade de São Paulo, São Paulo, São Paulo, Brazil; Miami University, United States of America

## Abstract

Hemotrophic mycoplasmas (hemoplasmas) are a group of animal pathogens of the *Mollicutes* class. Recently, the genomes of 8 hemoplasmas have been completely sequenced. The aim of this study was to gain a better understanding of their genomic features and relationship to other *Mycoplasma* species. The genome structure and dynamics of hemoplasmas were analyzed by evaluating gene synteny, adaptive evolution of paralogous gene families (PGF) and horizontal gene transfer (HGT). The *Mollicutes* class was then phylogenetically analyzed by constructing a distance matrix of the 16S rRNA genes and a phylogenetic tree with 32 conserved, concatenated proteins. Our results suggest that the hemoplasmas have dynamic genomes. The genome size variation (from 547 to 1,545 genes) indicates substantial gene gain/loss throughout evolution. Poorly conserved gene syntenies among hemoplasmas, positional shuffling of paralogous genes between strains, HGT, and codons under positive selection in PGFs were also observed. When compared to other *Mollicutes* species, the hemoplasmas experienced further metabolic reduction, and the 16S rRNA gene distance matrix of the available mollicutes suggests that these organisms presently constitute the most divergent clade within its class. Our phylogenetic tree of concatenated proteins showed some differences when compared to the 16S rRNA gene tree, but non-mycoplasma organisms, such as *Ureaplasma* spp. and *Mesoplasma* spp., continue to branch within *Mycoplasma* clades. In conclusion, while the hemoplasmas experienced further metabolic shrinkage through gene loss, PGFs with positively selected codons are likely beneficial to these species. Phylogeny of the mollicutes based on 16S rRNA genes or concatenated proteins do not obey the current taxonomy. The metabolism and genetic diversity of the mollicutes, the presence of HGT, and lack of standard for genus circumscription are likely to hinder attempts to classify these organisms based on phylogenetic analyses.

## Introduction

Members of the *Mollicutes* class are wall-less bacteria found in a wide range of host species, such as humans, mammals, fish, reptiles, arthropods and plants. The most studied genus within the class, the *Mycoplasma*, is known by its ample pathogenic potential, establishing commensal as well as highly virulent relationships with their hosts. As the smallest self-replicating organisms described to date, these organisms underwent an extensive genome reduction, with genome sizes varying from 580 Kb to 1.3 Mb. This reduction resulted in a concise metabolism with multifunctional enzymes, low genomic redundancy and complex regulation [1], [2]. However, despite this genetic minimalism, these organisms are capable of surviving within the hosts for extended periods of time, often evading the immune responses and establishing chronic infection.

In the early 1900's, several similarly small, uncultivable organisms were described attached to the erythrocytes of mammals. Due to their small size, gram-negative staining, tropism for erythrocytes and putative arthropod transmission, these bacteria were classified within the *Anaplasmataceae* family, *Ricketsialles* order, as *Eperythrozoon* spp. and *Haemobartonella* spp. [3]. However, in 1997 the 16S rRNA genes of some of these organisms (*Eperythrozoon suis*, *E. wenyonii*, *Haemobartonella felis*, *H. muris*) were sequenced and phylogenetically analyzed [4]. Surprisingly, these bacteria appeared closely related to the pneumoniae group of the *Mycoplasma* genus. In 2001, there was an official proposal to transfer these organisms to the *Mycoplasmataceae* family as *Mycoplasma* spp. [5]. Today, these bacteria comprise a distinct cluster of erythrocyte-associated organisms within a group that was mostly known for the colonization of mucosal and/or joint epithelia.

The close evolutionary relationship of the 16S rRNA genes between the hemoplasmas and other mycoplasma species, in particular the pneumoniae group, was convincing evidence to reclassify these organisms within the *Mycoplasmataceae* family. However, the unique tropism for erythrocytes, as well as relatively low sequence similarity when compared to the closest related mucosal mycoplasma species (between 77 and 83%) raised concerns regarding the appropriateness of the hemoplasmas allocation to the genus *Mycoplasma* [6], [7]. In the last edition of the Bergey's Manual, these bacteria were then placed in the *Mycoplasmatales* family *incertae sedis*, which serve to group taxonomic clades from which broader relationships are unknown or undefined.

In the past years, despite their lack of *in vitro* cultivation, the genomes of six hemotrophic mycoplasma species were sequenced [8]–[15]. These sequences provided novel information about the hemoplasmas pathogenicity mechanisms, metabolism and most importantly, divergences when compared to other mycoplasma species. Taken together, these characteristics motivated further studies regarding the genetics of the hemoplasmas. The aim of this study was to gain a better understanding of genomic features of the hemoplasmas and their relationship to other *Mycoplasma* species.

## Materials and Methods

### Genome sequences and proteomes

The 53 members of the *Mollicutes* class included in this study, including all eight hemoplasmas sequenced to date, are listed in Table S1. For the evaluation of horizontal gene transfer, one representative proteome of each mollicutes species was used.

### Gene synteny

Whole genome synteny, i.e. gene order, was compared between species using CoGe (genomevolution.org/CoGe) and Sybil [16], [17]. In CoGe, SynMap was used to generate two-dimensional dot-plot synteny maps and regions of interested were manually inspected using GEvo (Genome Evolution Analysis). SynMap uses DAGchainer algorithm coupled with BLASTp to identify syntenic homologous proteins; each dot represents putative homologous genes between any two genomes (www.genomevolution.org/wiki).

In Sybil, orthologs (homologous protein sequences from different bacterial species) were used to define syntenic relationships between species/strains. Sybil and associated algorithms identify clusters of homologous protein sequences using reciprocal best BLAST [18] match corrected for paralogs (homologous protein sequences within the same bacterial genome or species) as previously described [16], [19]. Briefly, an all-vs-all BLASTp search identified pairs of best-hit genes within each genome of the hemoplasmas (parameters: e-value of 1e-05, 80% identity, 70% coverage cutoffs). This hit graph was used to identify paralogous genes through the Jaccard algorithm with a cutoff of 0.6. Paralog clusters were then organized into ortholog clusters by allowing any member of a paralog cluster to contribute to the reciprocal best matches used to construct the ortholog clusters. Synteny plots were then built by coloring CDSs of a query/reference genome in a gradient from yellow to blue, left to right. If the query genome shares an ortholog cluster with another genome, this cluster is indicated above the reference sequences using the color that corresponds to the query CDS position in its native genome [19].

### Characterization and positive selection of paralogous gene families (PGF)

PGFs of hemoplasmas were identified and grouped using BLASTClust (30% identity and 70% coverage cutoffs) [9]. The number of PGFs and genome sizes were correlated using simple linear regression analysis, and proteins separated into functional categories according to TIGR roles (www.tigr.org).

Positive selection of PGFs was evaluated using algorithms available at Datamonkey, a web-server of the HyPhy package [20]. This analysis indentifies codons under positive or negative selection within each PGF alignment by estimating the relative rates of synonymous and non-synonymous substitutions. For our purposes, positive selection was defined as a significant excess of non-synonymous (resulting in an amino-acid change) over synonymous (not resulting in an amino-acid change) nucleotide substitutions. When present, this evolutionary pressure is believed to confer an advantageous genetic trait for the gene family. Briefly, individual nucleotide sequences of each PGF (>8 members, which is the minimum number of nucleotide sequences required by the Datamonkey algorithms) were aligned using MUSCLE (codons) [21], [22] and corrected for recombination using GARD (genetic algorithm for recombination detection) with general discrete site-to-site variation and 2 rate classes [23]. Recombination-corrected alignments were then analyzed using fixed effects likelihood (FEL) method for the identification of positively selected codon-sites [20] with general reversible (REV) nucleotide substitution bias model and posterior probability >0.05. Thirty randomly selected conserved bacterial genes were used as control of the same analysis procedure (Table S2).

### Pan- and core-genome of hemoplasmas

Pan- and core-genome plots of hemoplasmas were constructed as previously described [24]. The number of genes was represented as a function of the number of sequenced genomes. Error bars were constructed to represent the 1^st^ and 3^rd^ quartile of these samples, and diamonds (core-genome plot) and triangles (pan-genome plot) were added to represent the medians. The power law function was then fit to all medians.

### Cluster of orthologous groups of *Mollicutes*

A total of 35 proteomes of mollicutes (Table S1), including 6 hemoplasma species, each representing one different bacterial species downloaded from NCBI, were included in this analysis. OrthoMCL software was used to identify clusters of orthologous groups (COGs) among these proteomes [25]. Only proteins longer than 30 amino acids were included. Briefly, homologous pairs of sequences were found using the all-against-all BLASTp algorithm with an E-value <1e-4. OrthoMCL then converted the BLASTp results into a normalized similarity matrix that was analyzed by a Markov Cluster algorithm (MCL) for clustering of orthologous sequences. The inflation index of 1.5 was used to regulate cluster tightness.

### Metabolic pathways

Previous analyses of hemoplasmas' metabolisms [9], [11], [13] and KEGG (Kyoto Encyclopedia of Genes and Genomes) [26] pathway database were used to compare different metabolic pathways among hemoplasma species and members of the *Mollicutes* class.

### Horizontal gene transfer (HGT)

COGs containing at least one hemoplasma (COGh) species identified with the OrthoMCL software were selected for HGT analyses. In order to detect gene gain or loss, the “presence” or “absence” of genes from COGh was mapped onto the leaves of a 16S rRNA phylogenetic tree of mollicutes using the modular system Mesquite v.2.75 [27]. Briefly, 16S rRNA gene sequences of all 35 organisms were aligned using MUSCLE [21] and a phylogenetic tree constructed using the neighbor-joining method [28], with Kimura 2-parameter and 1,000 bootstrap replicates from MEGA 5 [22]. This tree was loaded into Mesquite v.2.75 and the presence or absence of each COGh gene was mapped onto its leaves. Ancestral state was predicted using Maximum Likelihood [29]. The pattern was then defined as a loss event if the gene was present in the ancestral node and one descendant node but absent in another node. Conversely, the pattern was defined as a gain event if the gene was absent in the ancestral node and one descendant node but was present in another descendant node [30]. Since gain events are the ones associated with HGT [30], COGh showing at least one gene gain event were selected for further analyses.

One hemoplasma representative protein sequence from within each gain-associated COGh was analyzed using BLASTp against nr (non-redundant protein sequences) database. An e-value cutoff of 1e-5 was used and results were filtered for protein length coverage (more than 50%). The first 100 hits were then retrieved for phylogenetic reconstruction. These sequences were aligned using MUSCLE [21] and phylogenetic trees were constructed using neighbor-joining [28] with 1,000 bootstrap replicates in MEGA 5 [22]. The resulting trees were manually compared to the 16S rRNA phylogenetic tree to infer HGT as described previously [30]. To be considered a true event, it had to be strongly supported by >50% bootstrap values [30]. Non-orthologous groups of hemoplasmas were analyzed in the same manner.

### Phylogenetic analyses

In order to evaluate 16S rRNA gene similarity among *Mollicutes* species, pairwise distances were computed using the distance module with Kimura 2-parameter from MEGA 5 software [22]. Further phylogenetic analyses of the 53 mollicutes were also performed using a multiple sequence alignment of 32 concatenated protein sequences from each organism. These proteins were chosen based on previous reports of phylogenomic analyses in prokaryotes (Table S3) [31]–[33] according to their presence in all selected species, absence of additional fused domains, no subjection to HGT, and completeness [32]. Following protein concatenation using the UNION tool from EMBOSS [34], a multiple sequence alignment was created using MUSCLE [21]. The resulting alignment was employed to build a phylogenetic tree using the neighbor-joining method [28] and maximum likelihood, with 1,000 bootstrap replicates from MEGA 5 [22].

## Results and Discussion

### General features of hemoplasmas genomes

The major genomic features of the hemoplasmas are shown in Table 1. All hemoplasmas presented with a single, circular chromosome, except for ‘*Candidatus* Mycoplasma haemominutum’, which showed a putative linear chromosome. Interestingly, the number of CDSs varied from 547 to 1,545, suggesting substantial gene loss and/or gain throughout evolution. More than half of the CDSs had unknown function and were mostly unique to the hemoplasmas, which highlights the divergence of these species when compared to closely related mycoplasma organisms [9], [11]. Moreover, a great proportion of CDSs were organized into PGFs (24.5–72.4%), with *M. haemofelis* strains showing the highest percentages of paralogous CDSs (71.5–72.4%).

**Table 1:** General features of the hemoplasma genomes.

	*M. suis* Illinois	*M. suis* KI3809	*M. haemofelis* Ohio2	*M. haemofelis* Langford1	*M. haemocanis* Illinois	*M. wenyonii* Massachussets	*M. haemolamae* Purdue	*M. haemoninutum* Birmingham 1
**Genome (bp)**	742,431	709,270	1,155,937	1,147,259	919,992	650,228	756,845	513,880
**G+C (%)**	31.1	31.1	38.8	38.9	35.3	33.9	39.3	35.5
**Total genes**	880	829	1,583	1,580	1,207	687	961	582
**CDSs**	845	794	1,524	1,545	1,173	652	925	547
**Average CDS length (bp)**	780	777	696	705	726	855	699	867
**CDSs with predicted function**	293	271	299	317	286	281	280	219
	(34.7%)	(34.1%)	(19.6%)	(20.5%)	(24.4%)	(43.1%)	(30.3%)	(40%)
**Hypothetical CDSs**	552	523	1,225	1,228	887	371	645	328
	(65.3%)	(65.9%)	(80.4%)	(79.5%)	(75.%)	(56.9%)	(69.7%)	(60%)
**Pseudogenes**	4	15	25	NA	NA	NA	NA	NA
**CDS in PGF**	361	297	1,103	1,104	748	263	454	134
	(42.8%)	(37.4%)	(72.4%)	(71.5%)	(63.8%)	(40.3%)	(49.1%)	(24.5%)
**CDS in PGF (Hypothetical)**	328	265	1,072	1,075	726	235	430	132
	(90.9%)	(89.2%)	(97.2%)	(97.3%)	(97.1%)	(89.3%)	(94.7%)	(98.5%)
**CDS in PGF (w/function)** *	33	32	31	29	22	28	24	2
	(9.1%)	(10.8%)	(2.8%)	(2.7%)	(2.9%)	(10.7%)	(5.3%)	(1.5%)
**No of tRNAs**	32	32	31	31	31	32	33	32
**No of rRNA**								
**16S**	1	1	1	1	1	1	1	1
**23S**	1	1	1	1	1	1	1	1
**5S**	1	1	1	1	1	1	1	1

CDS: Coding DNA sequence; NA: not available.

* Paralogs with predicted function: ABC transporters, restriction-modification systems, ATP synthase subunits, DNA binding proteins, DNA and RNA polymerase subunits, DNA helicase, elongation factor Tu (2 copies in *M. wenyonii*) and aldo/keto reductase family proteins. Number of PGFs per genome: 68 in *M. suis* Illinois, 64 in ‘*Candidatus* M. haemolamae’, 51 in *M. wenyonii*, 45 in *M. haemofelis* strain Ohio2, 34 in *M. haemocanis* and 22 in ‘*Candidatus* M. haemominutum’.

The hemoplasmas can be phylogenetically separated in two clusters (suis and haemofelis) based on the presence of a 24 bp deletion on the 16S rRNA gene of the haemofelis members [4]. Interestingly, our study showed different rRNA genes organization and distribution of hypothetical *versus* non-hypothetical CDSs between these two groups. Organisms from the suis group (*M. suis*, *M. wenyonii*, ‘*Candidatus* M. haemolamae’, ‘*Candidatus* M. haemoninutum’) showed a 16S rRNA gene separated from the 23S-5S operon and non-hypothetical CDSs (mainly involved in metabolism) located near the origin (Ori) and terminus (Ter) of replication, whereas hypothetical proteins of PGF were situated away from these sites [9]. Conversely, organisms from the haemofelis group (*M. haemofelis*, *M. haemocanis*) showed all three rRNA genes organized in the same operon and non-hypothetical CDSs located only at the Ori, with PGFs of hypothetical proteins scattered throughout the genome [11], [13]. Also, these two latter organisms presented with larger genomes (919 kb–1.1 Mb) when compared to hemoplasmas in the suis group (513–756 Kb). Further genomes of the haemofelis group (e.g. *M. coccoides*, *M. haemomuris*, ‘*Candidatus* M. turicensis’) should be sequenced in order to confirm if these differences in gene organization, and genome architecture and size are in fact cluster-associated.

The number of pseudogenes appeared to be low (<25) or not calculated for most of the hemoplasma genomes. However, this number may not be accurately known because many members of the PGFs of hypothetical proteins may be pseudogenes mis-annotated as functional genes due to the low sensitivity of the detection methods currently employed [35]. Whether or not these are functional or non-functional segments of DNA [36] is unknown.

### Gene synteny

**Figure 1:**
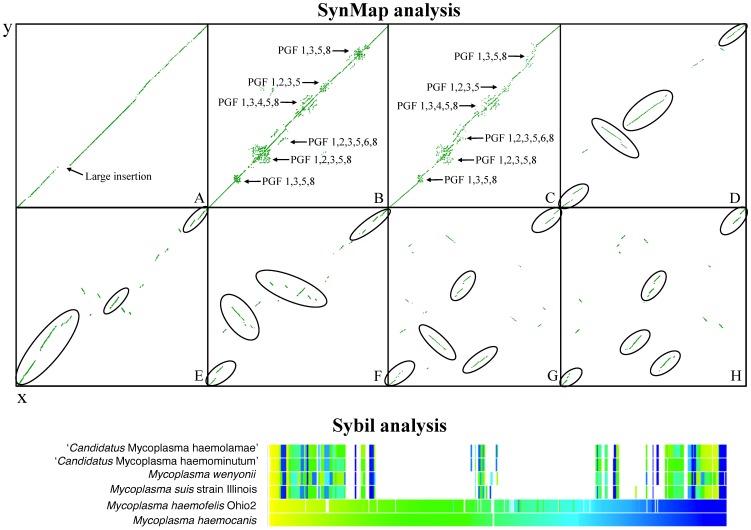
Syntenic maps of hemoplasma genomes. Plots were generated using comparative genomics suite CoGe SynMap and Sybil tool. In the SynMap analysis, each dot represents a matching gene pair. A) y-axis: *M. suis* Illinois, x-axis: *M. suis* KI3806; arrow indicates a 39,820 nt insertion in *M. suis* strain Illinois B) y-axis: *M. haemofelis* Langford1, x-axis: *M. haemofelis Ohio2* C) *M. haemocanis* Illinois x-axis: *M. haemofelis* Ohio2 D) y-axis: ‘*Candidatus* M. haemominutum’ Birmingham1, x-axis: *M. suis* Illinois E) y-axis: ‘*Candidatus* M. haemolamae’ Purdue, x-axis: ‘*Candidatus* M. haemominutum’ Birmingham1 F) y-axis: ‘*Candidatus* M. haemolamae’ Purdue, x-axis: *M. suis* Illinois G) y-axis: *M. wenyonii* Massachussets, x-axis: ‘*Candidatus* M. haemominutum’ Birmingham1 H) y-axis: *M. wenyonii* Massachussets, x-axis: *M. suis* Illinois. Other comparisons, which show less conserved synteny, are not shown for simplicity. Arrows indicate expansion of paralog gene families (PGF) as numbered in *M. haemofelis* Ohio2 [11]. Circles indicate blocks of synteny. Sybil map used *M. haemocanis* as a reference genome. See Materials and Methods.

Conserved synteny of genes has been commonly used as support for genome annotation and identification of orthologs [37], [38]. However, overall gene synteny is known to be lost at a much faster rate than sequence similarity throughout evolution [37] and it is thus not as frequently used to assess genome evolution, except in closely related bacterial strains. Accordingly, Sybil and SynMap analyses indicated a loss of overall gene synteny among hemoplasma species, with the exception of *M. haemofelis* and *M. haemocanis*. An inverse correlation between hemoplasma synteny and sequence divergence/phylogenetic relatedness could be observed (Figure 1). Although conserved blocks of genes, mostly corresponding to operons (e.g. phosphate ABC transporter: PstA, PstB, PstS), were detected using Sybil, they do not necessarily occur at the same relative position of the genomes (except for *M. haemofelis* and *M. haemocanis*), suggesting significant genomic reorganization (Figure 1).

As noted previously [13], *M. haemofelis* and *M. haemocanis* showed highly similar genome organization (Figure 1C). Areas of moderate genetic variability, found away from the Ori, were mostly related to hypothetical proteins of PGFs (Figure 1C). Due to the high identity of the 16S rRNA gene sequences of both species (>99%), *M. haemofelis* and *M. haemocanis* were once thought to be the same species infecting different hosts [39], [40]. However, based on prevailing standards for species circumscription using average nucleotide identity (ANI) and tetranucleotide analysis [41], these organisms can be currently classified as different species [13].

In contrast to the haemofelis group, only few collinear arrangements of genes involving mostly functional operons (data not shown) were present among members of the suis group (Figure 1D–H). Blocks of synteny, including inversions, were observed when comparing ‘*Candidatus* M. haemoninutum’ to *M. suis* Illinois and ‘*Candidatus* M. haemolamae’ and are represented by circles in Figure 1D,E.

SynMap pairwise comparisons showed highly conserved gene synteny between hemoplasma strains (both *M. suis* and *M. haemofelis* strains) (Figure 1A,B). Nevertheless, a large area of insertion in the *M. suis* strain Illinois, that mostly explains the difference in genome size between *M. suis* strains, was observed (nucleotide position: 162,968 to 202,787; total of 39,820 nucleotides). This genetic insertion is composed of hypothetical proteins from PGF #1 and #2. Overall, the *M. suis* strain Illinois had 11 areas of DNA insertion (511 to 39,820 bp) when compared to strain KI3806, and strain KI3806 had 5 areas of DNA insertion (2,350 to 11,070 bp) when compared to strain Illinois. Most of these insertions were composed of hypothetical proteins within PGFs, except for 2 areas of insertion in *M. suis* strain Illinois, which contained HdsR (MSU_0811, 0812, 0813) and DpnII (MSU_0849) genes.

In contrast to *M. suis* strains, parallel diagonal lines representing six areas of PGFs of hypothetical proteins were observed between *M. haemofelis* strains Ohio2 and Langford1 (Figure 1B). These areas represent extensive gene shuffling related to these families, which was not observed in PGFs of *M. suis* strains. Reasons for this increased dynamism of the *M. haemofelis* genomes are unknown. A detailed comparison of insertions/deletions between *M. haemofelis* strains was reported elsewhere [11].

### PGF characterization

The most striking feature of the hemoplasmas genomes is the presence of numerous and large PGFs. Between 22 and 68 PGFs per genome varying from 2 to 800 members each were found among the hemoplasma species. This incredibly high number of 800 members in a PGF occurs in *M. haemofelis* strain Ohio2 and is possibly the largest PGF ever found in a prokaryote [11]. As observed previously with other bacterial genomes [42], the genome size of hemoplasmas strongly correlated with the number of paralogs in linear regression (R2 = 0.982) (Figure S1). The proportion of paralogous proteins within each hemoplasma also increased with their genome size (Table 1). Thus, PGF duplication events account for the genome size variation of hemoplasmas throughout evolution.

Previous analyses of bacterial PGFs indicated paralog retention bias towards specific functional classes [42]. *M. pneumoniae*, *M. genitalium*, *M. pulmonis* and *Ureaplasma urealyticum* possess a greater number of paralogs categorized as defense proteins (i.e multidrug efflux pumps, restriction-modification systems, etc) when compared to other functional categories. Although defense proteins were among the hemoplasma paralogs with known function (Table 1), most of their paralogs are hypothetical proteins (4,263/4,464; 95.5%).

### Adaptive evolution of PGFs

In order to evaluate if PGFs undergo adaptive evolution, we searched for evidence of positive selection. A total of 65.1% (41/63) of the analyzed PGFs of hemoplasmas were under positive selection in at least one codon site, varying from 1 to 7 positively selected sites per PGF (Table 2). This result suggests that the maintenance of diversity at these codon sites is an advantageous trait and these duplicated genes are likely to be beneficial within the bacterial population. Duplicated genes under positive selection have been also described in other blood pathogens, such as *Rickettsia* spp., *Trypanosoma brucei* and *Plasmodium vivax* [43]–[45]. The majority of these genes express surface proteins in interaction with the host environment. As few of these PGFs of *M. suis* and *M. haemofelis* have been found to encode immunogenic proteins [46] (Guimaraes et al., unpublished data), rapid amino acid changes are likely to be crucial for function and for evading the host immune defenses. Therefore, while host-dependency contributes to genome shrinkage through relaxation of positive selection, other selective pressures, e.g. host immune response [47], may act on PGFs and lead to the maintenance of their codon diversity. It is still unknown, however, if these families are contracting or expanding overtime.

**Table 2:** Positive selection analysis of paralogous gene families (PGFs) of hemoplasmas.

		PGFs	
Hemoplasma	No of PGFs*	PGFs under positive selection (range of positive sites/PGF) (range of negative sites/PGF)	PGFs under negative selection only (range of negative sites/PGF)
*M. suis* Illinois	14	12 (1–6) (23–127)	2 (31,35)
*M. haemofelis* Ohio2	8	4 (1–3) (34–120)	4 (39–185)
*M. haemocanis*	9	5 (1–3) (22–146)	4 (68–119)
*M. haemolamae*	20	13 (1–3) (32–157)	7 (32–72)
*M. haemominutum*	4	3 (1–2) (58–100)	1 (115)
*M. wenyonii*	8	4 (1–7) (28–91)	4 (31–183)
Total	63	41 (1–7) (22–157)	22 (31–183)
Controls (Table S2)		No positive selection	Multiple negative sites

* Only PGF with >8 members were tested. Positive sites: codons that were identified as being positively selected. Negative sites: codons that were identified as being negatively selected.

### Pan- and core-genome of hemoplasmas

The pan- and core-genome plots of the hemoplasmas are shown in Figure S2. The pan-genome represents the cumulative number of COGs present in all hemoplasma genomes, while the core-genome represents the conserved number of COGs. Accordingly, the pan-genome of the hemoplasmas was composed of approximately 1,474 genes. As more hemoplasma genomes were compared, the number of COGs of the pan-genome did not reach a plateau line (saturation), which indicates that a larger pool of hemoplasma genes still remains to be discovered. These genes will most likely be species-specific. On the contrary, the number of shared COGs (core-genome) suggested a finite number of approximately 244 genes (15.5% to 41.9% of the genes of any hemoplasma isolate). Considering that most of these genes have known functions and are distributed among different functional categories (see below), the basic genetic pool of the hemoplasmas, as a group, is already known.

### COGs of the *Mollicutes*

**Figure 2:**
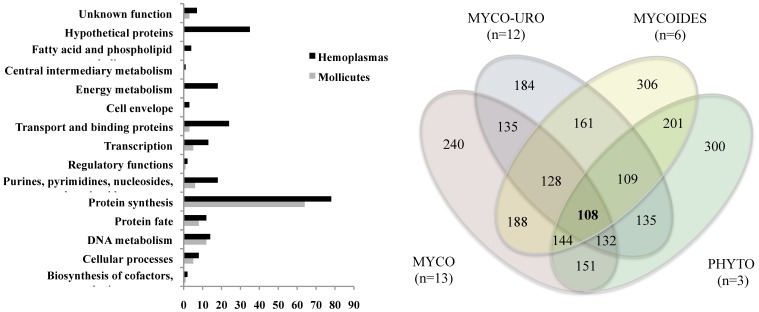
Cluster of orthologous groups (COG) analysis of *Mollicutes* species. A) Functional categories comparison of the COGs of *Mollicutes* and hemoplasmas. B) COGs of *Mollicutes* (n = 108) and its phylogenetic clades (MYCO: Mycoplasma clade; MYCO-URO: Mycoplasma-Ureaplasma clade; MYCOIDES: Mycoides-Entomoplasmataceae cluster; PHYTO: Phytoplasma clade. Phylogenetic groups were defined as described by Volokhov et al. [62]. The hemoplasmas are part of the Mycoplasma-Ureaplasma clade). X-axis indicate the number of COGs.

The number of COGs between any two hemoplasma species varied from 295 to 319, except for *M. haemofelis* strains and *M. haemocanis*, which varied from 935 to 944 (Table S4). When considering all 8 hemoplasma genomes, only 236 COGs were identified. As expected [2], most of these 236 COGs had known functions (non-hypothetical proteins: 201, 85.2%; hypothetical proteins: 35, 14.8%) spread throughout 13 different functional categories (Figure 2). These COGs included only from 15.78% (*M. haemofelis* Ohio2) to 43.69% (‘*Candidatus* M. haemominutum’) of all proteins of each hemoplasma genome. On the other hand, the majority of the non-orthologous proteins of hemoplasmas had unknown function (hypothetical proteins) (from *M. wenyonii* with 84.89%, 336/416, to ‘*Candidatus* M. haemominutum’ with 94.21%, 293/311) and were mostly part of PGFs [from ‘*Candidatus* M. haemominutum” with 45.05% (132/293) to *M. haemofelis* Langford with 90.11% (1075/1190)].

These small numbers of hemoplasma COGs, the great proportion of hypothetical non-orthologous proteins, and the lack of gene synteny among different hemoplasma species likely correspond to the introduction of hypothetical proteins and/or PGFs during speciation events of the hemoplasma group. These speciation events may be associated with evolutionary host shifts, e.g hemoplasma species originating from a common ancestor that now infect different host species, leading to the gain or loss of unique sets of genes for each hemoplasma species. The exception lies with *M. haemofelis* and *M. haemocanis*; very similar organisms (with 935–944 orthologs), infecting different hosts and causing different disease presentations.

The COGs of *Mollicutes* and its phylogenetic clades are shown in Figure 2. Only 108 COGs were found to be common among all *Mollicutes* species, and none of these clusters were composed of hypothetical proteins. In contrast to the hemoplasmas, the majority of these clusters covered only 8 functional categories, being the majority related to protein synthesis (59%; e.g. ribosomal proteins), DNA metabolism (11.1%; e.g. DNA gyrase) and protein fate (7.4%; e.g. Sec). Other important functional categories, such as energy metabolism and transport, varied greatly among the *Mollicutes* species, possibly due to gene loss. Therefore, this COG analysis suggests that the *Mollicutes* class is a highly diverse group of organisms in regard to their metabolic functions. Only few functional categories were conserved among all species. Its phylogenetic clades (i.e hemoplasmas), on the other hand, showed an increased number of conserved COGs that covered most functional categories (Figure 2). This finding suggests that the phylogenetic clades have more conserved metabolic functions than all 53 members of the *Mollicutes* class taken together.

### Metabolic pathways

Further analysis of these functional categories indicated that the hemoplasmas have fairly conserved metabolic pathways. Comparative analyses revealed minimal metabolism variation, with only three metabolic pathways showing disparities among the hemoplasma species. In the nicotinate/nicotinamide pathway, the enzyme NAD^+^ kinase was absent in all hemoplasmas, except *M. haemofelis* and *M. haemocanis*. Interestingly, NAD^+^ kinase-coding genes were also not identified in *Plasmodium* species [48]. It is thus unknown how these organisms produce NADP^+^. Also, the enzyme nicotinate phosphoribosyltransferase was only present in *M. suis*, ‘*Candidatus* M. haemominutum’ and ‘*Candidatus* M. haemolamae’. Since other enzymes from this pathway were missing, the preferred source of NAD^+^ (nicotinate or nicotinamide) cannot be predicted.

In the pyrimidine metabolism, cytidylate kinase was also only present in *M. haemofelis* and *M. haemocanis*. In organisms where cytidylate kinase was absent, it had been proposed that deoxycytidine or cytidine 5′triphosphate (dCTP or CTP) is produced from uracil, instead of cytosine, through the use of the multifunctional enzyme phosphofructokinase [9]. However, even in *M. haemofelis* and *M. haemocanis*, certain enzymes from the pathway that converts cytosine into dCTP or CTP were missing. Therefore, it is unknown if dCTP/CTP is produced from cytosine and/or uracil residues in hemoplasmas. Recently, cytidylate kinase has been described to have a positive impact on the efficiency of nucleotide synthesis in *Corynebacterium glutamicum* under microaerobic conditions [49]. Whether or not the presence of this enzyme favors the survival of *M. haemofelis* and *M. haemocanis* in oxygen-limited environments needs to be further explored.

In the purine metabolism, hypoxanthine phosphoribosyltransferase was only present in *M. haemofelis*, *M. haemocanis* and *M. wenyonii*. This enzyme catabolizes the reaction from hypoxanthine to IMP (inosine 5′-monophosphate), which serves as precursor for purine nucleotides. Even though this enzyme was absent in other hemoplasmas, a partial hypoxanthine phosphoribsyltransferase domain fusioned to the adenylossucinate lyase enzyme (e.g., gene id: MSU_0708) is likely to exert its function. Another possibility would be to import and utilize IMP.

When compared to other mollicutes, the hemoplasmas have experienced further metabolic reduction. They lost all the enzymes of the pentose-phosphate (PP) pathway, pyruvate dehydrogenase complex (PDC) and coenzyme-A (coA) metabolism. Among *Mollicutes* species, the complete absence of the PP pathway has been described only in *Phytoplasma* spp and it has been connected to life in a nutrient rich environment [50]. In other organisms, some enzymes of this pathway are missing, but the pathway is functional, producing ribose from glucose [1]. In addition, the lack of the PDC hampers the hemoplasmas' ability to generate additional energy through the oxidation of pyruvate to acetyl-CoA. Likewise, this complex was not detected in members of the hominis and ureaplasma clusters. The absence of the coA metabolism goes along with this PDC absence, as this complex utilizes coA as a cofactor. Consequences of the absence of proteins and enzymes associated with the coA metabolism on the lipid synthesis are unknown. The loss of these genes is another evidence of genome minimization towards a highly efficient metabolism.

Two interesting differences of hemoplasmas compared to the majority of *Mollicutes* species were the utilization of hypoxanthine in the purine metabolism and the presence of the enzyme NADP-dependent glyceraldehyde 3-phosphate dehydrogenase (GAPN). As hypoxanthine is the most common purine in the blood, it has been speculated that its pathway is an adaption to life in the blood environment [9]. *M. penetrans* and *Mesoplasma florum* were the only mollicutes found to have an identical pathway to hemoplasmas, yet they are not closely related to these organisms. *M. mycoides*, *M. iowae*, *Acholeplasma laidlawii*, *M. putrefaciens* showed only similar pathways. Therefore, few mollicutes have the ability to use hypoxanthine to synthesize purine nucleotides. It is likely that gene loss and/or gain was evolutionarily tailored according to nutrient availability.

The GAPN enzyme was present in only nine *Mollicutes* species sequenced to date, including 5 species from the pneumoniae group (*M. penetrans*, *M. iowae*, *U. parvum*, *U. urealyticum*, *M. gallisepticum*, *M. leachii*, *M. myocides*, *S. melliferum*, *M. florum*) scattered throughout different phylogenetic clusters (muris, pneumoniae, mycoides, spiroplasma clusters). GAPN catalyzes the oxidation of glyceraldehyde-3-phosphate to 3-phosphoglycerate using the reduction of NADP+ to NADPH, possibly preserving the production of NADPH in the absence of the PP pathway [51], [52]. GAPN is also known to be resistant to hydrogen peroxide, which is likely present in the blood environment [53].

### Horizontal gene transfer

Until recently, it was thought that HGT in mycoplasma species and other symbiotic or parasitic organisms was a rare event [54]–[56]. However, there is increasing evidence that variable proportions of mycoplasma genomes have undergone HGT [57], [58]. In order to search for such events, we analyzed all proteins from mollicutes (Table S1) using OrthoMCL. Details about the selection of COGs, phylogenetic reconstructions and analysis of non-orthologous proteins are described in Supplementary Material S1. Only six (6 out of 64; 9.37%) COGs/genes showed phylogenetic support for putative HGT (Table 3, Figures S5, S6, S7, S8, S9).

**Table 3:** Putative gene candidates with phylogenetic support for HGT.

Gene ID	Annotation	Putative Recipient Species	Putative Donor Species	Phylogenetic tree (Figure)
WEN_00280	Hypoxanthine phosphoribosyltransferase type I	*M. wenyonii*	*Babesia equi*	S5
MHF_0096, MHC_00425	Hypoxanthine phosphoribosyltransferase type II^1^	*M. haemofelis*, *M. haemocanis*	*M. penetrans*	S5
MHC_05205, HF1_14200, MHF_1490	ATP-dependent DNA helicase UvrD/PcrA	*M. haemocanis*, *M. haemofelis* 2	*Eubacterium rectale*	S6
MHC_05210	Hypothetical protein^3^	Hemoplasmas	*Ureaplasma* spp., *M. penetrans*, *M. iowae*	S7
MHC_ 05495, MHF_1548	pfkB family kinase	*Babesia equi*	*M. haemofelis* and *M. haemocanis*	S8
MHF_0804, MHC_02800	Hypothetical protein	*M. haemocanis*, *M. haemofelis*	Mycoplasma clade or Pneumoniae-genitalium cluster	S9

1 Two similar proteins annotated as hypoxanthine phosphoribosyltranferase are observed in *M. haemofelis* and *M. haemocanis* (MHF_0096, 0098 and MHC_00425, 00435; 24–26% identity, 475 to 498 amino acids long). It is possible that MHF_0096 and MHC_00425 are the *Mollicutes*-related genes and were not horizontally transferred; while MHF_0098 and MHC_00435, homologs to WEN_00280, were horizontally acquired, as demonstrated for WEN_00280.

2 Three copies of this gene were observed in *M. haemofelis* (Ohio2 and Langford1) and 4 copies were observed in *M. haemocanis*; only the genes listed were associated with HGT.

3 There are two copies of this gene in *M. haemofelis* and *M. haemocanis*; therefore, there is possibility of gene duplication and the results should be evaluated with caution.

There were other 18 cases in which positional shuffling of members from the suis group was observed in the phylogenetic reconstruction, often suggesting the presence of several donor/recipient species and possible HGT (Table S5). However, a great proportion (10; 55.6%) of these phylogenetic reconstructions had borderline bootstrap values (between 40 and 60). Since gene transfer is biased towards transfer between closely related organisms [59], HGT among hemoplasmas cannot be ruled out, but further validation is necessary. Whether or not this shuffling occurs by chance due to high identity among protein sequences or it represents actual transfer events is unknown. The method used herein is more suitable for detection of HGT among more distantly related organisms [30]. And finally, there were 7 cases in which the external, donor and recipient species were all hemoplasma species, but the external and the recipient species were the same organism, e.g. *M. suis*, representing paralogous genes (Table S5). In such situations, it is not possible to distinguish between gene duplication (with posterior sequence divergence) and HGT.

In addition to the complete absence of BLAST hits that hampered HGT analysis, reasons for low bootstrap values in the phylogenetic reconstruction or absence of HGT detection include: low protein length coverage and/or presence of gaps, gene duplication that hampered phylogenetic reconstruction (e.g. HsdS genes), and insufficient number of BLAST hits that did not allow detection of recipient/donor/external species. Therefore, given the high stringency of the method used and still low number of homologous proteins in the NCBI databases, it is likely that the number of horizontally transferred genes is much higher than observed herein. In particular, the enzyme superoxide dismutase (SOD), which has been detected only in *M. haemofelis*, *M. haemocanis* and *M. iowae*, was not identified as a horizontally transferred gene due to low bootstrap values in the phylogenetic reconstruction with the current BLAST hits. In the future, the availability of additional gene sequences in the NCBI databases may facilitate detection.

The molecular mechanism by which the hemoplasmas are able to transfer genes is unknown. Nevertheless, the horizontal transfer of genes related to metabolism, .e.g. hypoxanthine phosphoribosyltransferase, highlights the usage of this mechanism to adapt into a different environment, the blood. More interesting is the fact that *Babesia* species, eukaryotic organisms that target the same host cell, are involved in this exchange. Gene transfer between prokaryotes and eukaryotes has been previously described [60], [61] and it is mostly associated to close interactions between bacteria and eukaryotes in a symbiotic fashion or environmentally.

### Phylogenetic analysis

The low sequence identity of the 16S rRNA gene of the hemoplasmas to their closest related mycoplasma organisms has been previously described [6]. Herein, we estimated evolutionary distances between sequences by computing the proportion of nucleotide differences between each pair of 16S rRNA genes of *Mollicutes* species. Values displayed on a distance table (Table S6) are inversely correlated to sequence identity. The hemoplasma cluster showed distances on the range of 0.23–0.37, the highest values observed among all analyzed mollicutes. Thus, the hemoplasmas appeared as the most divergent group within the *Mollicutes* class based on the 16S rRNA gene sequences. It is worth mentioning that this result is based on the 16S rRNA gene sequences available for this study. Future analyses should include additional members of *Acholeplasmatales*, *Entomoplasmatales* and *Anaeroplasmatales* to acquire a more in-depth view of such divergences.

The taxonomy of the *Mollicutes* class is an unresolved issue because the phenotypic characterization does not necessarily match the rRNA phylogeny. The availability of genome sequences provides the opportunity to use whole-genome information to generate phylogenetic trees. In this study, phylogenetic trees based on concatenated protein sequences were constructed. This approach has been frequently shown to increase resolution and robustness of phylogenetic analyses of prokaryote species [32]. Accordingly, the generated tree (Figure S3) showed similar topology to the 16S rRNA (Figure S4) gene tree, albeit with higher bootstrap values, and can be used in future studies where the phylogenetic analysis of 16S rRNA genes does not result in enough resolution. The difference in the positioning of groups between the 16S rRNA gene and the concatenated protein trees lies with the *Acholeplasma* and *Mycoides* clusters. In addition, the hemoplasma cluster showed a long branch, i.e. greater number of genetic changes, forming a strongly supported cluster separated from the genitalium-pneumoniae group. Non-mycoplasma organisms, such as *Ureaplasma* spp. and *Mesoplasma* spp., continue to branch within *Mycoplasma* clades and a revision of the *Mollicutes* class regarding genera classification is warranted in order to resolve this inconsistency.

## Conclusions

The hemoplasmas have highly dynamic genomes. Genome size variation, positional shuffling of genes related to PGFs, poorly conserved gene synteny and HGT are evidence of such dynamism. These organisms have experienced further metabolic reduction when compared to other mollicutes, but not all genes are in the path of reductionism. The detection of positive selection in PGFs suggests that codon diversity within these genes is an advantageous trait for the hemoplasmas and that these families are likely beneficial to these species.

The current taxonomy of the *Mollicutes* class is partially made based on phenotypic characteristics, and the predictive usefulness of it is considered high. These taxonomic characteristics, however, do not obey the phylogenetic classification based on the 16S rRNA gene sequences or on whole-genome information, possibly due to HGT. Ideally, this inconsistency should be resolved, but the lack of standards regarding genus circumscription (as it is available for species) and the presence of HGT are likely to hinder the “correct” classification of these organisms. The fact that the mollicutes are a metabolically and genetically diverse group, as exemplified by its low number of COGs and low sequence identity of the 16S rRNA genes among its members, may result in further taxonomic complications. And finally, until the hemoplasmas can be cultivated *in vitro* or there is a change for such requirement in the Bacteriological Code, these organisms will continue to be classified in the family *incertae sedis*.

## Supporting Information

Figure S1 **Number of protein coding sequences (CDSs) in paralogous gene families (PGFs) in function of the genome size of hemoplasmas.**
(PDF)

Figure S2 **Pan and core-genome plots of the hemoplasmas.**
(PDF)

Figure S3 **Phylogenetic trees based on 32 concatenated proteins of Mollicutes.**
(PDF)

Figure S4 **Phylogenetic tree based on 16S rRNA gene sequences of Mollicutes.**
(PDF)

Figure S5 **Phylogenetic tree of the horizontal gene transfer analysis (WEN_00280).**
(PDF)

Figure S6 **Phylogenetic tree of the horizontal gene transfer analysis (MHC_05205).**
(PDF)

Figure S7 **Phylogenetic tree of the horizontal gene transfer analysis (MHC_05210).**
(PDF)

Figure S8 **Phylogenetic tree of the horizontal gene transfer analysis (MHC_ 05495).**
(PDF)

Figure S9 **Phylogenetic tree of the horizontal gene transfer analysis (MHF_0804).**
(PDF)

Supplementary Material S1 **Horizontal gene transfer analysis.**
(PDF)

Table S1 **Bacterial organisms used in the horizontal gene transfer (HGT) and phylogeny studies.**
(DOCX)

Table S2 **Conserved bacterial genes used as control for the positive selection analysis.**
(DOCX)

Table S3 **Cluster of orthologous groups (COG)/proteins used in the phylogenetic analysis.**
(DOCX)

Table S4 **Number of Clusters of Orthologous Groups (COGs) between hemoplasma species.**
(DOCX)

Table S5 **Putative horizontally transferred genes among members of the suis groups and cases in which gene duplication could not be distinguished from HGT.**
(DOCX)

Table S6 **Estimates of Evolutionary Divergence between Sequences.**
(XLS)
