# Supplementary material for: Comparative Genomics and Phylogenomics of Hemotrophic Mycoplasmas
Source: PLoS One. 2014 Mar 18;9(3):e91445. doi: 10.1371/journal.pone.0091445 (PMC3958358; doi:10.1371/journal.pone.0091445)
Supplement: Figure S1 — Number of protein coding sequences (CDSs) in paralogous gene families (PGFs) in function of the genome size of hemoplasmas. (PDF) [file pone.0091445.s001.pdf]

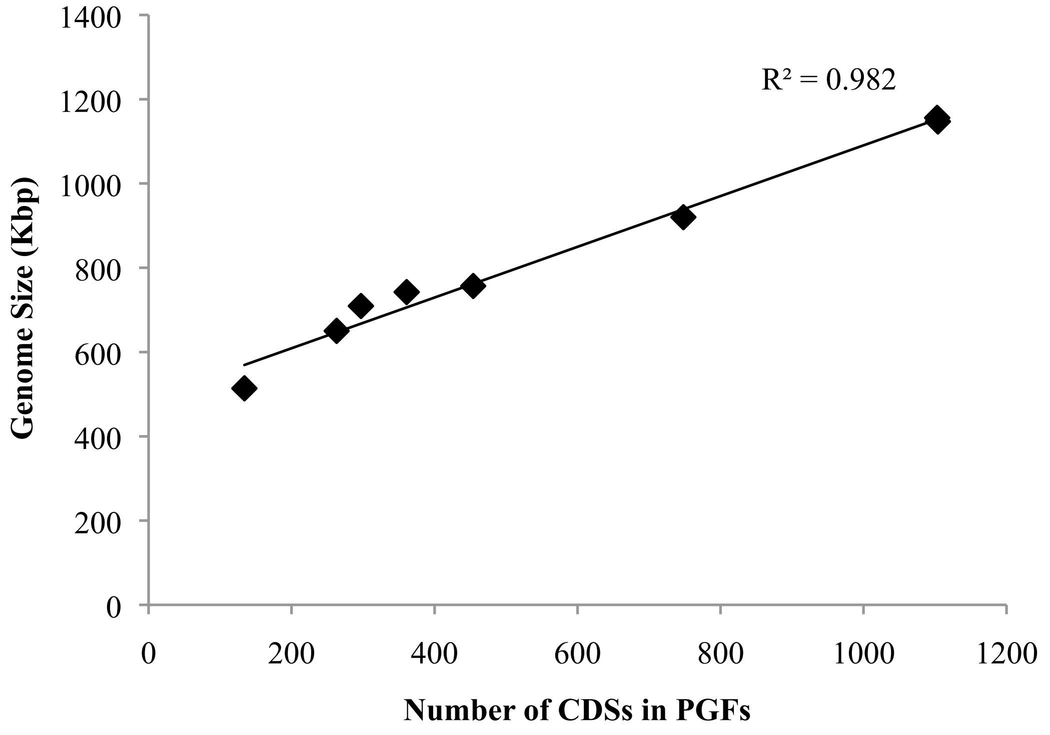

Figure S1. Number of protein coding sequences (CDSs) in paralogous gene families (PGFs) in function of the genome size of hemoplasmas. Simple linear regression was utilized.
