# Supplementary material for: Comparative Genomics and Phylogenomics of Hemotrophic Mycoplasmas
Source: PLoS One. 2014 Mar 18;9(3):e91445. doi: 10.1371/journal.pone.0091445 (PMC3958358; doi:10.1371/journal.pone.0091445)
Supplement: Figure S2 — Pan and core-genome plots of the hemoplasmas. (PDF) [file pone.0091445.s002.pdf]

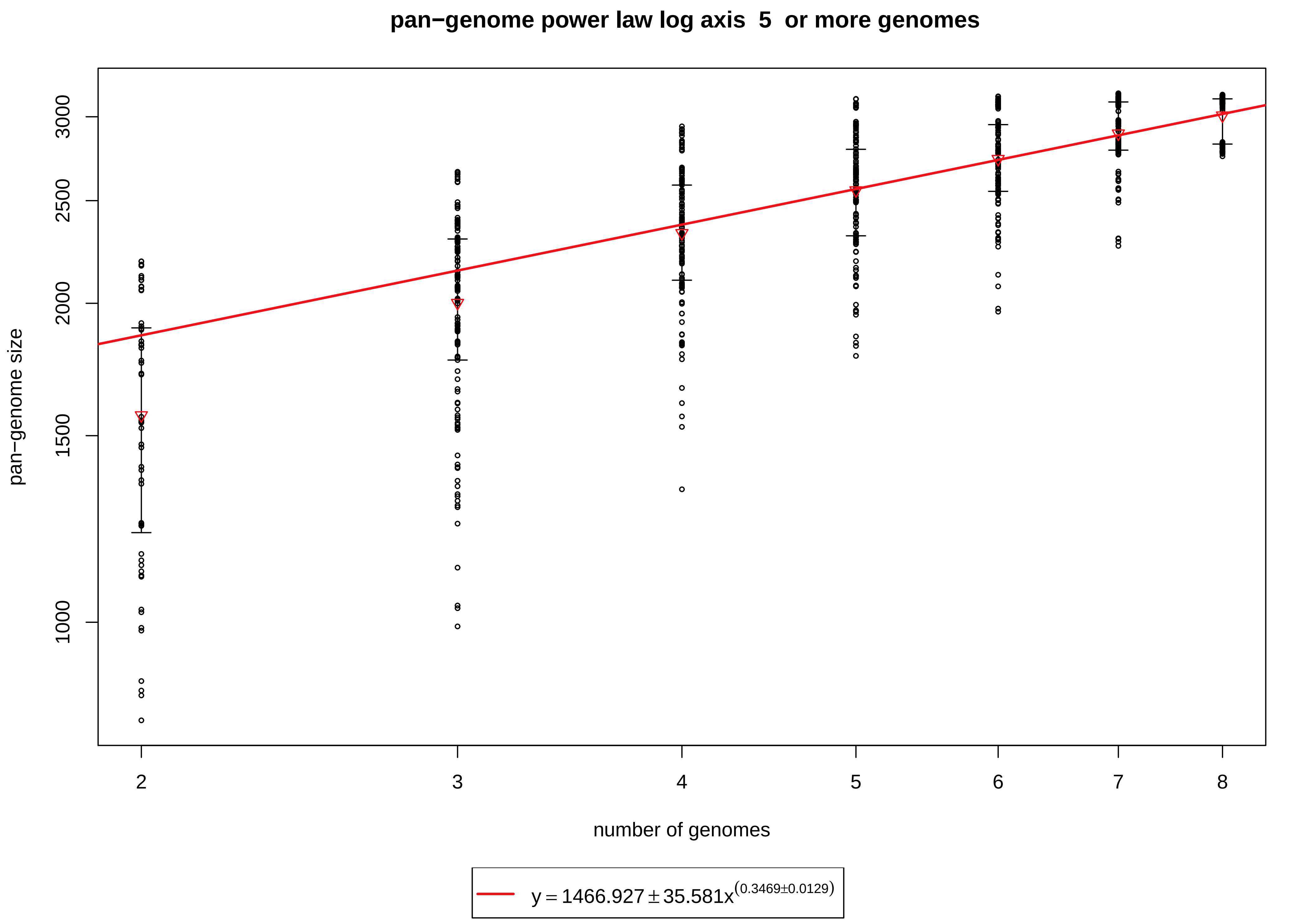

pan−genome power law log axis 5 or more genomes
pan−genome size
3000
2500
2000
1500
1000
2
3
4
5
6
7
8
number of genomes
y = 1466.927 ± 35.581x^(0.3469±0.0129)

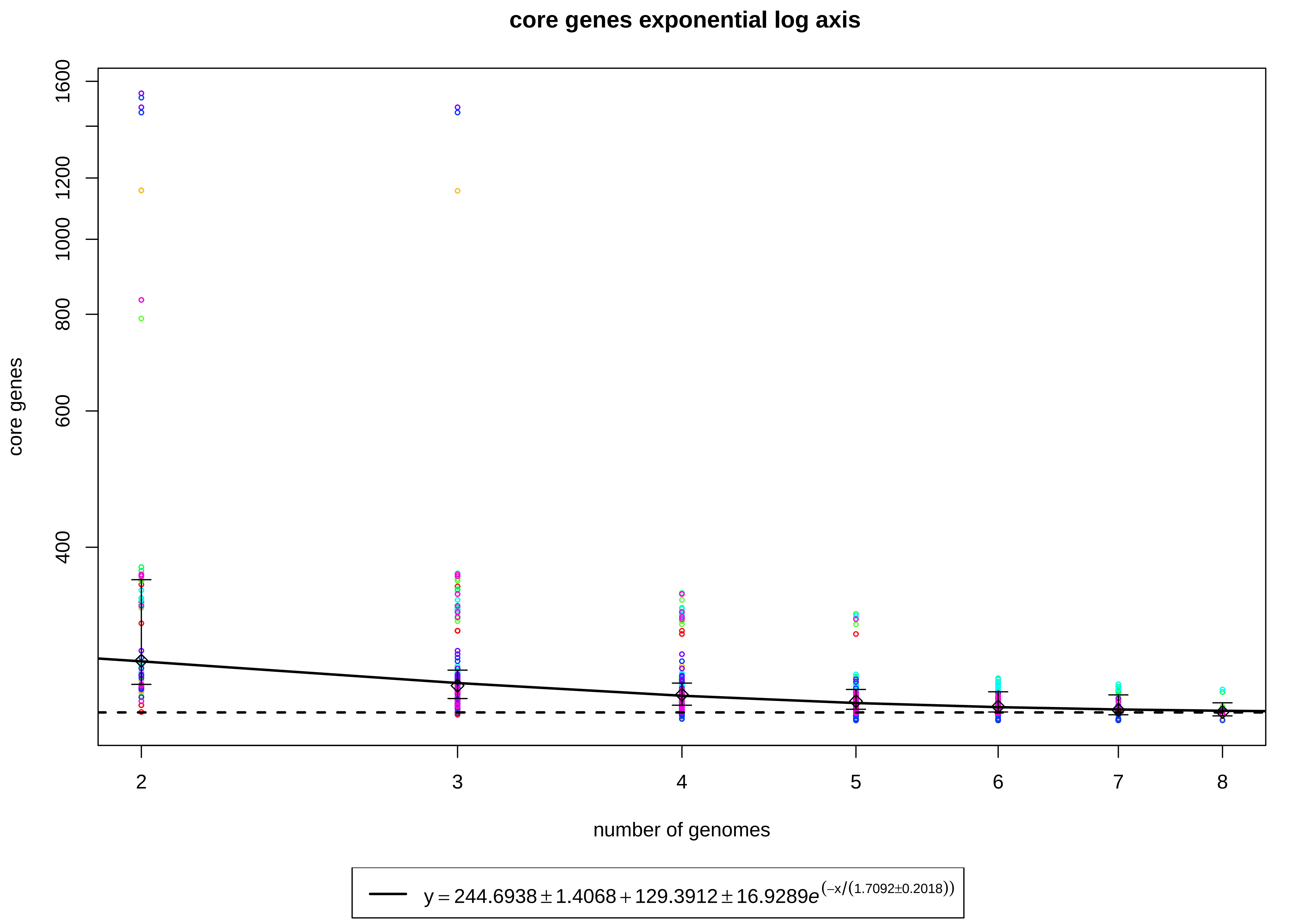
core genes exponential log axis
core genes
1600
1200
1000
800
600
400
2
3
4
5
6
7
8
number of genomes
$y = 244.6938 \pm 1.4068 + 129.3912 \pm 16.9289 e^{(-x/(1.7092 \pm 0.2018))}$

Figure S2. Pan and core-genome plots of the hemoplasmas. Plots were generated as previously described [24]. The number of genes is represented as a function of the number of sequenced genomes (x-axes: number of genomes; y-axes: number of genes). Genomes are sequentially sampled in all combinations until the last genome is analyzed. Error bars represent the 1st and 3rd quartile of these samples, and triangles (pan-genome plot) and diamonds (core-genome plot) represent the medians. The power law function is fit to all medians. The following genomes were used: *M. haemocanis* str. Illinois (PRJNA82367), *M. suis* str. Illinois (PRJNA61897), *M. haemofelis* strain Ohio2 (PRJNA162029), *M. wenyonii* str. Massachusetts (PRJNA168067), '*Candidatus* M. haemolamae' str. Purdue (PRJNA68151), '*Candidatus* M. haemominutum' str. Birmingham 1 (PRJNA76933), *M. suis* strain KI3806 (PRJNA63665), and *M. haemofelis* strain Langford1 (PRJNA62461).
