# Supplementary material for: Comparative Genomics and Phylogenomics of Hemotrophic Mycoplasmas
Source: PLoS One. 2014 Mar 18;9(3):e91445. doi: 10.1371/journal.pone.0091445 (PMC3958358; doi:10.1371/journal.pone.0091445)
Supplement: Figure S3 — Phylogenetic trees based on 32 concatenated proteins of Mollicutes. (PDF) [file pone.0091445.s003.pdf]

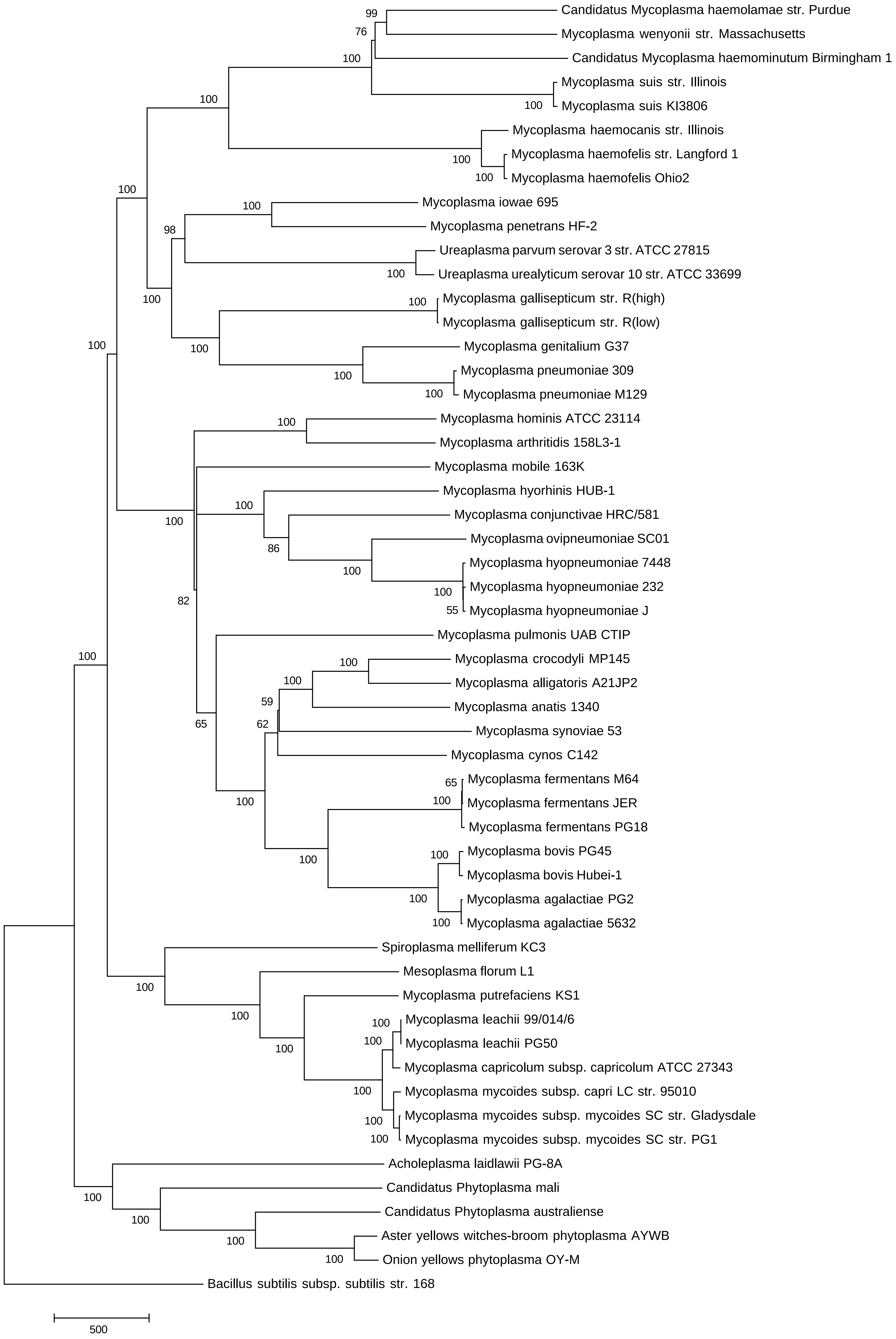

Candidatus Mycoplasma haemolamae str. Purdue
Mycoplasma wenyonii str. Massachusetts
Candidatus Mycoplasma haemominutum Birmingham 1
Mycoplasma suis str. Illinois
Mycoplasma suis KI3806
Mycoplasma haemocanis str. Illinois
Mycoplasma haemofelis str. Langford 1
Mycoplasma haemofelis Ohio2
Mycoplasma iowae 695
Mycoplasma penetrans HF-2
Ureaplasma parvum serovar 3 str. ATCC 27815
Ureaplasma urealyticum serovar 10 str. ATCC 33699
Mycoplasma gallisepticum str. R(high)
Mycoplasma gallisepticum str. R(low)
Mycoplasma genitalium G37
Mycoplasma pneumoniae 309
Mycoplasma pneumoniae M129
Mycoplasma hominis ATCC 23114
Mycoplasma arthritidis 158L3-1
Mycoplasma mobile 163K
Mycoplasma hyorhinis HUB-1
Mycoplasma conjunctivae HRC/581
Mycoplasma ovipneumoniae SC01
Mycoplasma hyopneumoniae 7448
Mycoplasma hyopneumoniae 232
Mycoplasma hyopneumoniae J
Mycoplasma pulmonis UAB CTIP
Mycoplasma crocodyli MP145
Mycoplasma alligatoris A21JP2
Mycoplasma anatis 1340
Mycoplasma synoviae 53
Mycoplasma cynos C142
Mycoplasma fermentans M64
Mycoplasma fermentans JER
Mycoplasma fermentans PG18
Mycoplasma bovis PG45
Mycoplasma bovis Hubei-1
Mycoplasma agalactiae PG2
Mycoplasma agalactiae 5632
Spiroplasma melliferum KC3
Mesoplasma florum L1
Mycoplasma putrefaciens KS1
Mycoplasma leachii 99/014/6
Mycoplasma leachii PG50
Mycoplasma capricolum subsp. capricolum ATCC 27343
Mycoplasma mycoides subsp. capri LC str. 95010
Mycoplasma mycoides subsp. mycoides SC str. Gladysdale
Mycoplasma mycoides subsp. mycoides SC str. PG1
Acholeplasma laidlawii PG-8A
Candidatus Phytoplasma mali
Candidatus Phytoplasma australiense
Aster yellows witches-broom phytoplasma AYWB
Onion yellows phytoplasma OY-M
Bacillus subtilis subsp. subtilis str. 168
500

Figure S3. Phylogenetic trees based on 32 concatenated proteins of *Mollicutes*. The tree was generated from a multiple sequence alignment using neighbor-joining (shown) and maximum likelihood (not shown) algorithms with 1,000 bootstrap replicates. *Bacillus subtillis* subsp. *subtillis* str. 168 was used as outgroup. All branches agree with those constructed using the maximum likelihood method. Bar shows substitution per nucleotide.
