# Supplementary material for: Comparative Genomics and Phylogenomics of Hemotrophic Mycoplasmas
Source: PLoS One. 2014 Mar 18;9(3):e91445. doi: 10.1371/journal.pone.0091445 (PMC3958358; doi:10.1371/journal.pone.0091445)
Supplement: Figure S4 — Phylogenetic tree based on 16S rRNA gene sequences of Mollicutes. (PDF) [file pone.0091445.s004.pdf]

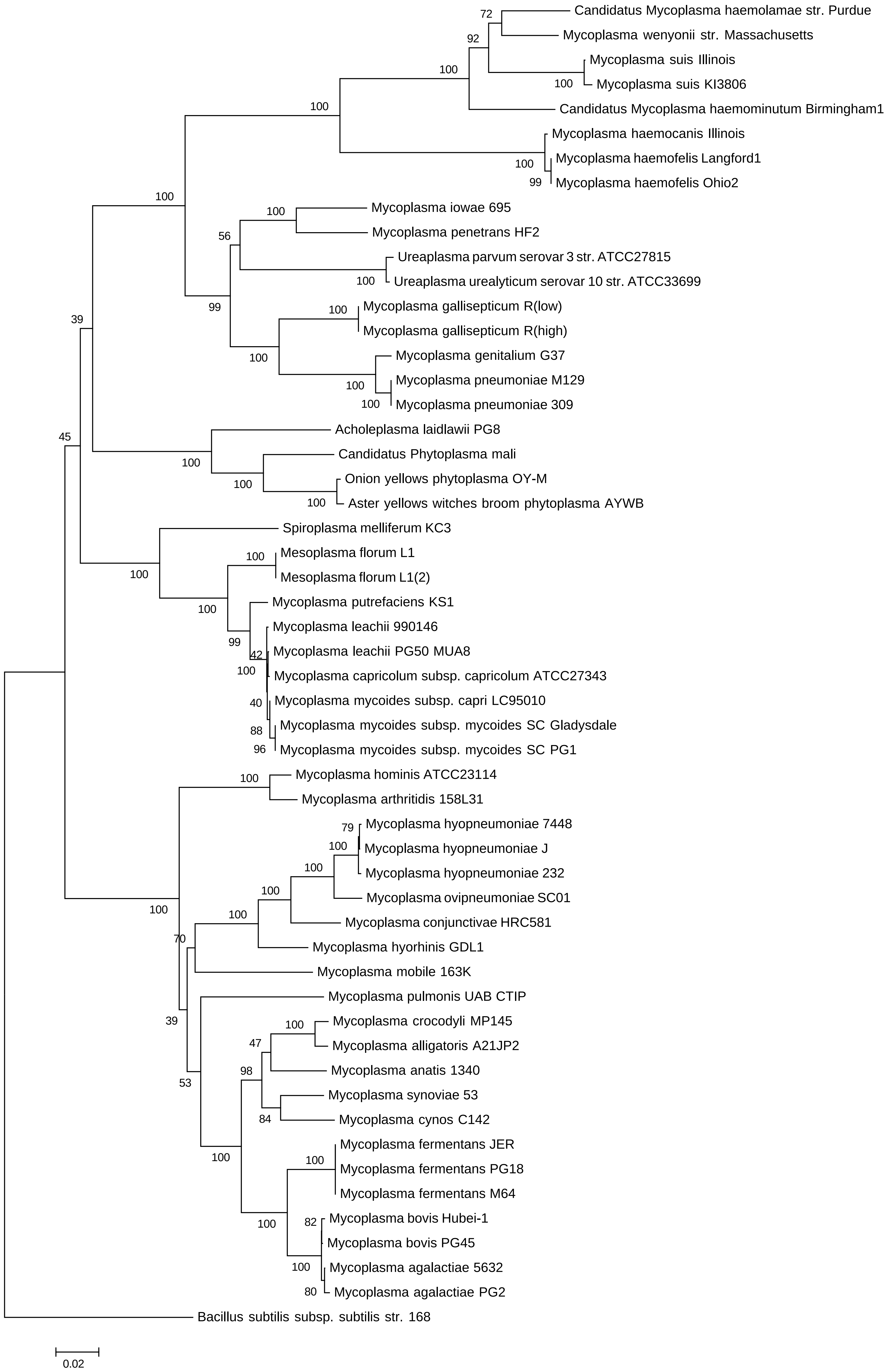

Candidatus Mycoplasma haemolamae str. Purdue
Mycoplasma wenyonii str. Massachusetts
Mycoplasma suis Illinois
Mycoplasma suis KI3806
Candidatus Mycoplasma haemominutum Birmingham1
Mycoplasma haemocanis Illinois
Mycoplasma haemofelis Langford1
Mycoplasma haemofelis Ohio2
Mycoplasma iowae 695
Mycoplasma penetrans HF2
Ureaplasma parvum serovar 3 str. ATCC27815
Ureaplasma urealyticum serovar 10 str. ATCC33699
Mycoplasma gallisepticum R(low)
Mycoplasma gallisepticum R(high)
Mycoplasma genitalium G37
Mycoplasma pneumoniae M129
Mycoplasma pneumoniae 309
Acholeplasma laidlawii PG8
Candidatus Phytoplasma mali
Onion yellows phytoplasma OY-M
Aster yellows witches broom phytoplasma AYWB
Spiroplasma melliferum KC3
Mesoplasma florum L1
Mesoplasma florum L1(2)
Mycoplasma putrefaciens KS1
Mycoplasma leachii 990146
Mycoplasma leachii PG50 MUA8
Mycoplasma capricolum subsp. capricolum ATCC27343
Mycoplasma mycoides subsp. capri LC95010
Mycoplasma mycoides subsp. mycoides SC Gladysdale
Mycoplasma mycoides subsp. mycoides SC PG1
Mycoplasma hominis ATCC23114
Mycoplasma arthritidis 158L31
Mycoplasma hyopneumoniae 7448
Mycoplasma hyopneumoniae J
Mycoplasma hyopneumoniae 232
Mycoplasma ovipneumoniae SC01
Mycoplasma conjunctivae HRC581
Mycoplasma hyorhinis GDL1
Mycoplasma mobile 163K
Mycoplasma pulmonis UAB CTIP
Mycoplasma crocodyli MP145
Mycoplasma alligatoris A21JP2
Mycoplasma anatis 1340
Mycoplasma synoviae 53
Mycoplasma cynos C142
Mycoplasma fermentans JER
Mycoplasma fermentans PG18
Mycoplasma fermentans M64
Mycoplasma bovis Hubei-1
Mycoplasma bovis PG45
Mycoplasma agalactiae 5632
Mycoplasma agalactiae PG2
Bacillus subtilis subsp. subtilis str. 168
0.02

Figure S4. Phylogenetic tree based on 16S rRNA gene sequences of *Mollicutes*. The tree was generated using neighbor-joining algorithm, with kimura-2 parameter and 1,000 bootstrap replications (MEGA 5 software). *Bacillus subtillis* subsp. *subtillis* str. 168 was used as outgroup. Bar shows substitution per nucleotide.
