# Supplementary material for: Comparative Genomics and Phylogenomics of Hemotrophic Mycoplasmas
Source: PLoS One. 2014 Mar 18;9(3):e91445. doi: 10.1371/journal.pone.0091445 (PMC3958358; doi:10.1371/journal.pone.0091445)
Supplement: Figure S5 — Phylogenetic tree of the horizontal gene transfer analysis (WEN_00280). (PDF) [file pone.0091445.s005.pdf]

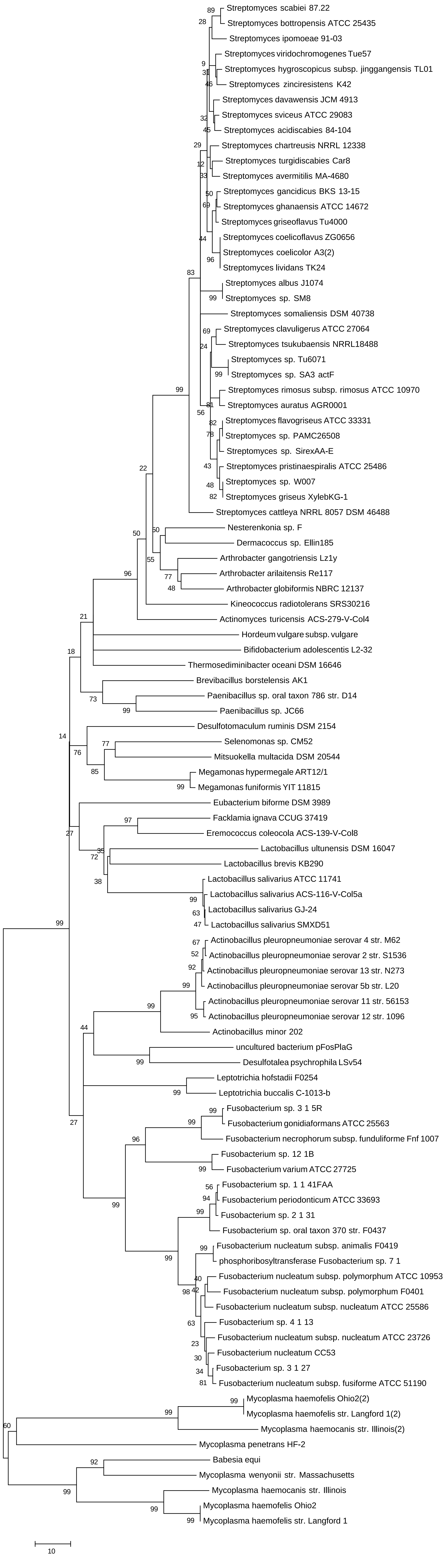
Streptomyces scabiei 87.22
Streptomyces bottropensis ATCC 25435
Streptomyces ipomoeae 91-03
Streptomyces viridochromogenes Tue57
Streptomyces hygroscopicus subsp. jinggangensis TL01
Streptomyces zincíresistens K42
Streptomyces davawensis JCM 4913
Streptomyces sviceus ATCC 29083
Streptomyces acidiscabies 84-104
Streptomyces chartreusis NRRL 12338
Streptomyces turgidiscabies Car8
Streptomyces avermitilis MA-4680
Streptomyces gancidicus BKS 13-15
Streptomyces ghanaensis ATCC 14672
Streptomyces griseoflavus Tu4000
Streptomyces coelicoflavus ZG0656
Streptomyces coelicolor A3(2)
Streptomyces lividans TK24
Streptomyces albus J1074
Streptomyces sp. SM8
Streptomyces somaliensis DSM 40738
Streptomyces clavuligerus ATCC 27064
Streptomyces tsukubaensis NRRL18488
Streptomyces sp. Tu6071
Streptomyces sp. SA3 actF
Streptomyces rimosus subsp. rimosus ATCC 10970
Streptomyces auratus AGR0001
Streptomyces flavogriseus ATCC 33331
Streptomyces sp. PAMC26508
Streptomyces sp. SirexAA-E
Streptomyces pristinaespiralis ATCC 25486
Streptomyces sp. W007
Streptomyces griseus XylebKG-1
Streptomyces cattleya NRRL 8057 DSM 46488
Nesterenkonia sp. F
Dermacoccus sp. Ellin185
Arthrobacter gangotriensis Lz1y
Arthrobacter arilaitensis Re117
Arthrobacter globiformis NBRC 12137
Kineococcus radiotolerans SRS30216
Actinomyces turicensis ACS-279-V-Col4
Hordeum vulgare subsp. vulgare
Bifidobacterium adolescentis L2-32
Thermosediminibacter oceani DSM 16646
Brevibacillus borstelensis AK1
Paenibacillus sp. oral taxon 786 str. D14
Paenibacillus sp. JC66
Desulfotomaculum ruminis DSM 2154
Selenomonas sp. CM52
Mitsuokella multacida DSM 20544
Megamonas hypermegale ART12/1
Megamonas funiformis YIT 11815
Eubacterium biforme DSM 3989
Facklamia ignava CCUG 37419
Eremococcus coleocola ACS-139-V-Col8
Lactobacillus ultunensis DSM 16047
Lactobacillus brevis KB290
Lactobacillus salivarius ATCC 11741
Lactobacillus salivarius ACS-116-V-Col5a
Lactobacillus salivarius GJ-24
Lactobacillus salivarius SMXD51
Actinobacillus pleuropneumoniae serovar 4 str. M62
Actinobacillus pleuropneumoniae serovar 2 str. S1536
Actinobacillus pleuropneumoniae serovar 13 str. N273
Actinobacillus pleuropneumoniae serovar 5b str. L20
Actinobacillus pleuropneumoniae serovar 11 str. 56153
Actinobacillus pleuropneumoniae serovar 12 str. 1096
Actinobacillus minor 202
uncultured bacterium pFosPlaG
Desulfotalea psychrophila LSv54
Leptotrichia hofstadii F0254
Leptotrichia buccalis C-1013-b
Fusobacterium sp. 3 1 5R
Fusobacterium gonidiaformans ATCC 25563
Fusobacterium necrophorum subsp. funduliforme Fnf 1007
Fusobacterium sp. 12 1B
Fusobacterium varium ATCC 27725
Fusobacterium sp. 1 1 41FAA
Fusobacterium periodonticum ATCC 33693
Fusobacterium sp. 2 1 31
Fusobacterium sp. oral taxon 370 str. F0437
Fusobacterium nucleatum subsp. animalis F0419
phosphoribosyltransferase Fusobacterium sp. 7 1
Fusobacterium nucleatum subsp. polymorphum ATCC 10953
Fusobacterium nucleatum subsp. polymorphum F0401
Fusobacterium nucleatum subsp. nucleatum ATCC 25586
Fusobacterium sp. 4 1 13
Fusobacterium nucleatum subsp. nucleatum ATCC 23726
Fusobacterium nucleatum CC53
Fusobacterium sp. 3 1 27
Fusobacterium nucleatum subsp. fusiforme ATCC 51190
Mycoplasma haemofelis Ohio2(2)
Mycoplasma haemofelis str. Langford 1(2)
Mycoplasma haemocanis str. Illinois(2)
Mycoplasma penetrans HF-2
Babesia equi
Mycoplasma wenyonii str. Massachusetts
Mycoplasma haemocanis str. Illinois
Mycoplasma haemofelis Ohio2
Mycoplasma haemofelis str. Langford 1
10

Figure S5. Phylogenetic tree of the horizontal gene transfer analysis. This tree is the phylogenetic reconstruction of the 100 first hits retrieved from the BLAST search using WEN_00280 protein sequence (hypoxanthine phosphoribosyltransferase type I, *M. wenyonii*) (Table 3). Sequences were aligned using MUSCLE [21] and the tree constructed using neighbor-joining [28] with 1,000 bootstrap replicates in MEGA 5 [22]. This tree was manually compared to a 16S rRNA gene phylogenetic tree to infer HGT as previously described [30].
