# Supplementary material for: Comparative Genomics and Phylogenomics of Hemotrophic Mycoplasmas
Source: PLoS One. 2014 Mar 18;9(3):e91445. doi: 10.1371/journal.pone.0091445 (PMC3958358; doi:10.1371/journal.pone.0091445)
Supplement: Figure S6 — Phylogenetic tree of the horizontal gene transfer analysis (MHC_05205). (PDF) [file pone.0091445.s006.pdf]

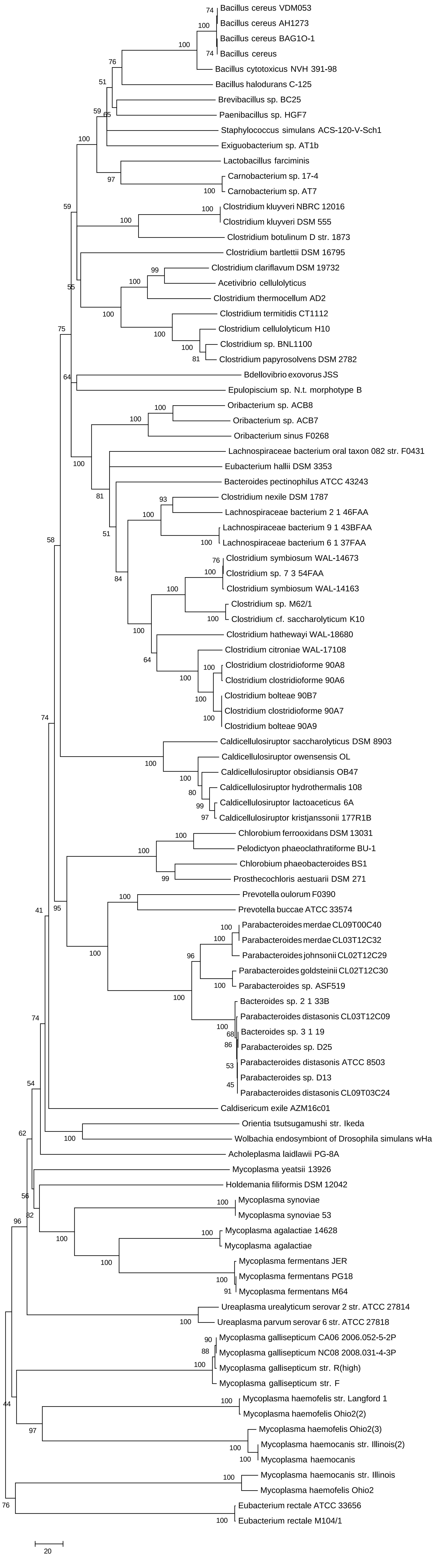
Bacillus cereus VDM053
Bacillus cereus AH1273
Bacillus cereus BAG1O-1
Bacillus cereus
Bacillus cytotoxicus NVH 391-98
Bacillus halodurans C-125
Brevibacillus sp. BC25
Paenibacillus sp. HGF7
Staphylococcus simulans ACS-120-V-Sch1
Exiguobacterium sp. AT1b
Lactobacillus farciminis
Carnobacterium sp. 17-4
Carnobacterium sp. AT7
Clostridium kluyveri NBRC 12016
Clostridium kluyveri DSM 555
Clostridium botulinum D str. 1873
Clostridium bartlettii DSM 16795
Clostridium clariflavum DSM 19732
Acetivibrio cellulolyticus
Clostridium thermocellum AD2
Clostridium termitidis CT1112
Clostridium cellulolyticum H10
Clostridium sp. BNL1100
Clostridium papyrosolvens DSM 2782
Bdellovibrio exovorus JSS
Epulopiscium sp. N.t. morphotype B
Oribacterium sp. ACB8
Oribacterium sp. ACB7
Oribacterium sinus F0268
Lachnospiraceae bacterium oral taxon 082 str. F0431
Eubacterium hallii DSM 3353
Bacteroides pectinophilus ATCC 43243
Clostridium nexile DSM 1787
Lachnospiraceae bacterium 2 1 46FAA
Lachnospiraceae bacterium 9 1 43BFAA
Lachnospiraceae bacterium 6 1 37FAA
Clostridium symbiosum WAL-14673
Clostridium sp. 7 3 54FAA
Clostridium symbiosum WAL-14163
Clostridium sp. M62/1
Clostridium cf. saccharolyticum K10
Clostridium hathewayi WAL-18680
Clostridium citroniae WAL-17108
Clostridium clostridioforme 90A8
Clostridium clostridioforme 90A6
Clostridium bolteae 90B7
Clostridium clostridioforme 90A7
Clostridium bolteae 90A9
Caldicellulosiruptor saccharolyticus DSM 8903
Caldicellulosiruptor owensensis OL
Caldicellulosiruptor obsidiansis OB47
Caldicellulosiruptor hydrothermalis 108
Caldicellulosiruptor lactoaceticus 6A
Caldicellulosiruptor kristjanssonii 177R1B
Chlorobium ferrooxidans DSM 13031
Pelodictyon phaeoclathratiforme BU-1
Chlorobium phaeobacteroides BS1
Prosthecochloris aestuarii DSM 271
Prevotella oulorum F0390
Prevotella buccae ATCC 33574
Parabacteroides merdae CL09T00C40
Parabacteroides merdae CL03T12C32
Parabacteroides johnsonii CL02T12C29
Parabacteroides goldsteinii CL02T12C30
Parabacteroides sp. ASF519
Bacteroides sp. 2 1 33B
Parabacteroides distasonis CL03T12C09
Bacteroides sp. 3 1 19
Parabacteroides sp. D25
Parabacteroides distasonis ATCC 8503
Parabacteroides sp. D13
Parabacteroides distasonis CL09T03C24
Caldisericum exile AZM16c01
Orientia tsutsugamushi str. Ikeda
Wolbachia endosymbiont of Drosophila simulans wHa
Acholeplasma laidlawii PG-8A
Mycoplasma yeatsii 13926
Holdemania filiformis DSM 12042
Mycoplasma synoviae
Mycoplasma synoviae 53
Mycoplasma agalactiae 14628
Mycoplasma agalactiae
Mycoplasma fermentans JER
Mycoplasma fermentans PG18
Mycoplasma fermentans M64
Ureaplasma urealyticum serovar 2 str. ATCC 27814
Ureaplasma parvum serovar 6 str. ATCC 27818
Mycoplasma gallisepticum CA06 2006.052-5-2P
Mycoplasma gallisepticum NC08 2008.031-4-3P
Mycoplasma gallisepticum str. R(high)
Mycoplasma gallisepticum str. F
Mycoplasma haemofelis str. Langford 1
Mycoplasma haemofelis Ohio2(2)
Mycoplasma haemofelis Ohio2(3)
Mycoplasma haemocanis str. Illinois(2)
Mycoplasma haemocanis
Mycoplasma haemocanis str. Illinois
Mycoplasma haemofelis Ohio2
Eubacterium rectale ATCC 33656
Eubacterium rectale M104/1
20

Figure S6. Phylogenetic tree of the horizontal gene transfer analysis. This tree is the phylogenetic reconstruction of the 100 first hits retrieved from the BLAST search using MHC_05205 protein sequence (ATP-dependent DNA helicase UvrD/PcrA, *M. haemocanis*) (Table 3). Sequences were aligned using MUSCLE [21] and the tree constructed using neighbor-joining [28] with 1,000 bootstrap replicates in MEGA 5 [22]. This tree was manually compared to a 16S rRNA gene phylogenetic tree to infer HGT as previously described [30].
