# Supplementary material for: Comparative Genomics and Phylogenomics of Hemotrophic Mycoplasmas
Source: PLoS One. 2014 Mar 18;9(3):e91445. doi: 10.1371/journal.pone.0091445 (PMC3958358; doi:10.1371/journal.pone.0091445)
Supplement: Figure S7 — Phylogenetic tree of the horizontal gene transfer analysis (MHC_05210). (PDF) [file pone.0091445.s007.pdf]

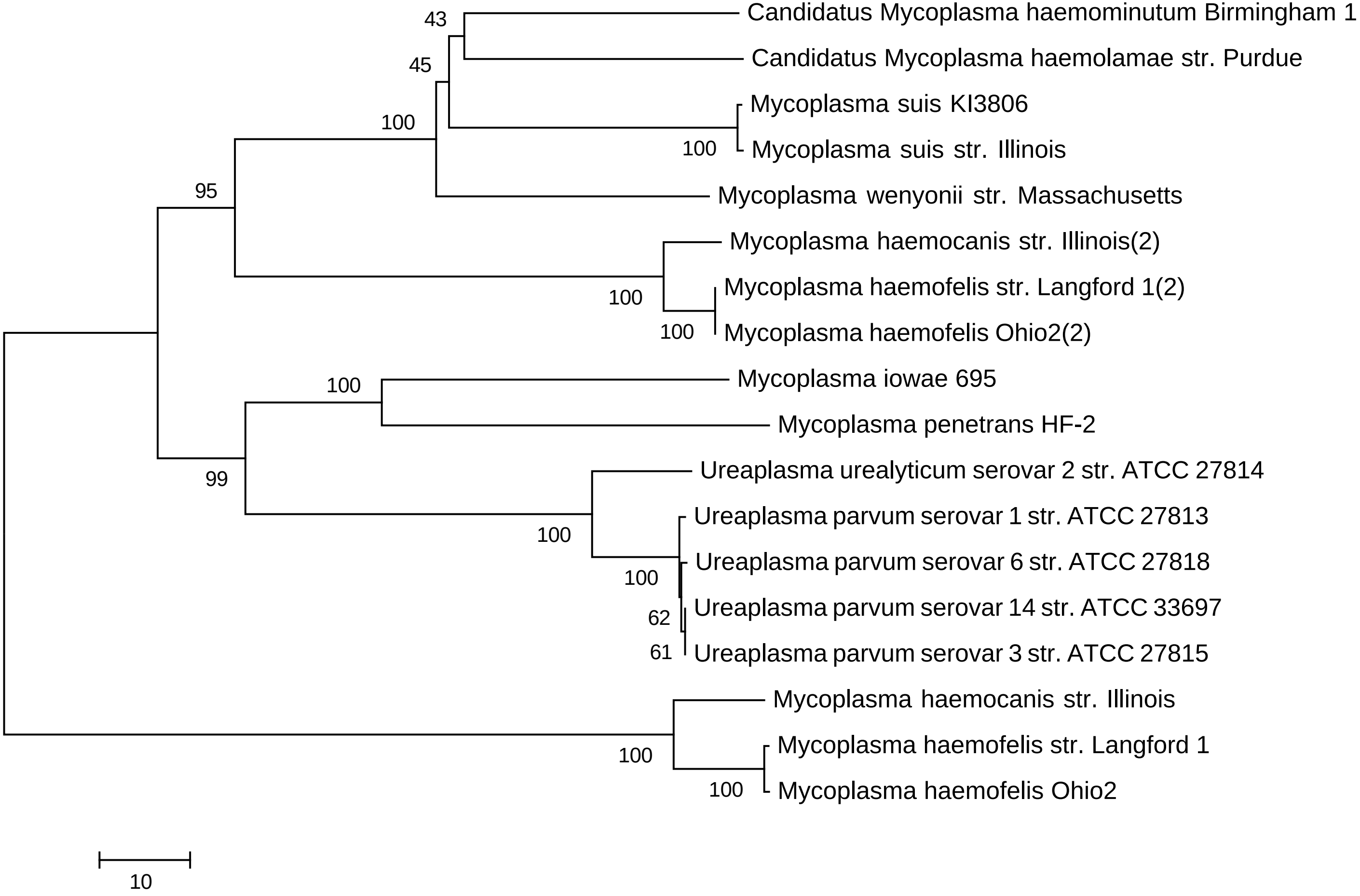
Candidatus Mycoplasma haemominutum Birmingham 1
Candidatus Mycoplasma haemolamae str. Purdue
Mycoplasma suis KI3806
Mycoplasma suis str. Illinois
Mycoplasma wenyonii str. Massachusetts
Mycoplasma haemocanis str. Illinois(2)
Mycoplasma haemofelis str. Langford 1(2)
Mycoplasma haemofelis Ohio2(2)
Mycoplasma iowae 695
Mycoplasma penetrans HF-2
Ureaplasma urealyticum serovar 2 str. ATCC 27814
Ureaplasma parvum serovar 1 str. ATCC 27813
Ureaplasma parvum serovar 6 str. ATCC 27818
Ureaplasma parvum serovar 14 str. ATCC 33697
Ureaplasma parvum serovar 3 str. ATCC 27815
Mycoplasma haemocanis str. Illinois
Mycoplasma haemofelis str. Langford 1
Mycoplasma haemofelis Ohio2
43
45
100
100
95
100
100
100
99
100
100
62
61
100
100
10

Figure S7. Phylogenetic tree of the horizontal gene transfer analysis. This tree is the phylogenetic reconstruction of the 100 first hits retrieved from the BLAST search using MHC_05210 protein sequence (hypothetical protein, *M. haemocanis*) (Table 3). Sequences were aligned using MUSCLE [21] and the tree constructed using neighbor-joining [28] with 1,000 bootstrap replicates in MEGA 5 [22]. This tree was manually compared to a 16S rRNA gene phylogenetic tree to infer HGT as previously described [30].
