# Supplementary material for: Comparative Genomics and Phylogenomics of Hemotrophic Mycoplasmas
Source: PLoS One. 2014 Mar 18;9(3):e91445. doi: 10.1371/journal.pone.0091445 (PMC3958358; doi:10.1371/journal.pone.0091445)
Supplement: Figure S8 — Phylogenetic tree of the horizontal gene transfer analysis (MHC_ 05495). (PDF) [file pone.0091445.s008.pdf]

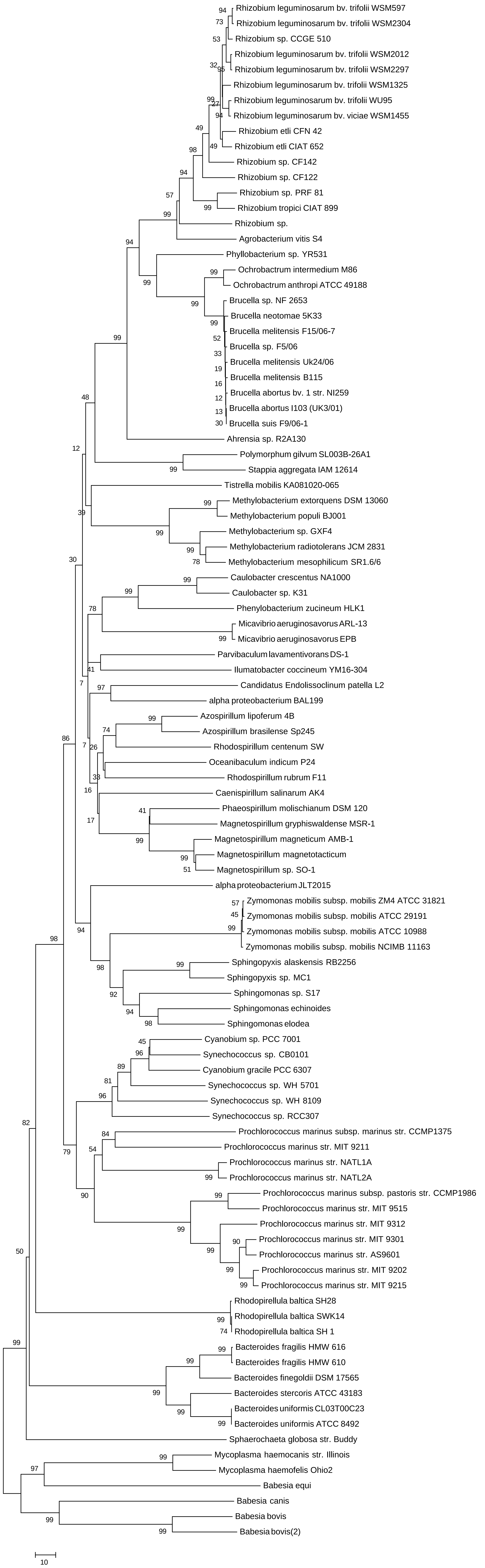
Rhizobium leguminosarum bv. trifolii WSM597
Rhizobium leguminosarum bv. trifolii WSM2304
Rhizobium sp. CCGE 510
Rhizobium leguminosarum bv. trifolii WSM2012
Rhizobium leguminosarum bv. trifolii WSM2297
Rhizobium leguminosarum bv. trifolii WSM1325
Rhizobium leguminosarum bv. trifolii WU95
Rhizobium leguminosarum bv. viciae WSM1455
Rhizobium etli CFN 42
Rhizobium etli CIAT 652
Rhizobium sp. CF142
Rhizobium sp. CF122
Rhizobium sp. PRF 81
Rhizobium tropici CIAT 899
Rhizobium sp.
Agrobacterium vitis S4
Phyllobacterium sp. YR531
Ochrobactrum intermedium M86
Ochrobactrum anthropi ATCC 49188
Brucella sp. NF 2653
Brucella neotomae 5K33
Brucella melitensis F15/06-7
Brucella sp. F5/06
Brucella melitensis Uk24/06
Brucella melitensis B115
Brucella abortus bv. 1 str. NI259
Brucella abortus I103 (UK3/01)
Brucella suis F9/06-1
Ahrensia sp. R2A130
Polymorphum gilvum SL003B-26A1
Stappia aggregata IAM 12614
Tistrella mobilis KA081020-065
Methylobacterium extorquens DSM 13060
Methylobacterium populi BJ001
Methylobacterium sp. GXF4
Methylobacterium radiotolerans JCM 2831
Methylobacterium mesophilicum SR1.6/6
Caulobacter crescentus NA1000
Caulobacter sp. K31
Phenylobacterium zucineum HLK1
Micavibrio aeruginosavorus ARL-13
Micavibrio aeruginosavorus EPB
Parvibaculum lavamentivorans DS-1
Ilumatobacter coccineum YM16-304
Candidatus Endolissoclinum patella L2
alpha proteobacterium BAL199
Azospirillum lipoferum 4B
Azospirillum brasilense Sp245
Rhodospirillum centenum SW
Oceanibaculum indicum P24
Rhodospirillum rubrum F11
Caenispirillum salinarum AK4
Phaeospirillum molischianum DSM 120
Magnetospirillum gryphiswaldense MSR-1
Magnetospirillum magneticum AMB-1
Magnetospirillum magnetotacticum
Magnetospirillum sp. SO-1
alpha proteobacterium JLT2015
Zymomonas mobilis subsp. mobilis ZM4 ATCC 31821
Zymomonas mobilis subsp. mobilis ATCC 29191
Zymomonas mobilis subsp. mobilis ATCC 10988
Zymomonas mobilis subsp. mobilis NCIMB 11163
Sphingopyxis alaskensis RB2256
Sphingopyxis sp. MC1
Sphingomonas sp. S17
Sphingomonas echinoides
Sphingomonas elodea
Cyanobium sp. PCC 7001
Synechococcus sp. CB0101
Cyanobium gracile PCC 6307
Synechococcus sp. WH 5701
Synechococcus sp. WH 8109
Synechococcus sp. RCC307
Prochlorococcus marinus subsp. marinus str. CCMP1375
Prochlorococcus marinus str. MIT 9211
Prochlorococcus marinus str. NATL1A
Prochlorococcus marinus str. NATL2A
Prochlorococcus marinus subsp. pastoris str. CCMP1986
Prochlorococcus marinus str. MIT 9515
Prochlorococcus marinus str. MIT 9312
Prochlorococcus marinus str. MIT 9301
Prochlorococcus marinus str. AS9601
Prochlorococcus marinus str. MIT 9202
Prochlorococcus marinus str. MIT 9215
Rhodopirellula baltica SH28
Rhodopirellula baltica SWK14
Rhodopirellula baltica SH 1
Bacteroides fragilis HMW 616
Bacteroides fragilis HMW 610
Bacteroides finegoldii DSM 17565
Bacteroides stercoris ATCC 43183
Bacteroides uniformis CL03T00C23
Bacteroides uniformis ATCC 8492
Sphaerochaeta globosa str. Buddy
Mycoplasma haemocanis str. Illinois
Mycoplasma haemofelis Ohio2
Babesia equi
Babesia canis
Babesia bovis
Babesia bovis(2)
10

Figure S8. Phylogenetic tree of the horizontal gene transfer analysis. This tree is the phylogenetic reconstruction of the 100 first hits retrieved from the BLAST search using MHC_ 05495 protein sequence (pfkB family kinase, *M. haemocanis*) (Table 3). Sequences were aligned using MUSCLE [21] and the tree constructed using neighbor-joining [28] with 1,000 bootstrap replicates in MEGA 5 [22]. This tree was manually compared to a 16S rRNA gene phylogenetic tree to infer HGT as previously described [30].
