# Supplementary material for: Comparative Genomics and Phylogenomics of Hemotrophic Mycoplasmas
Source: PLoS One. 2014 Mar 18;9(3):e91445. doi: 10.1371/journal.pone.0091445 (PMC3958358; doi:10.1371/journal.pone.0091445)
Supplement: Figure S9 — Phylogenetic tree of the horizontal gene transfer analysis (MHF_0804). (PDF) [file pone.0091445.s009.pdf]

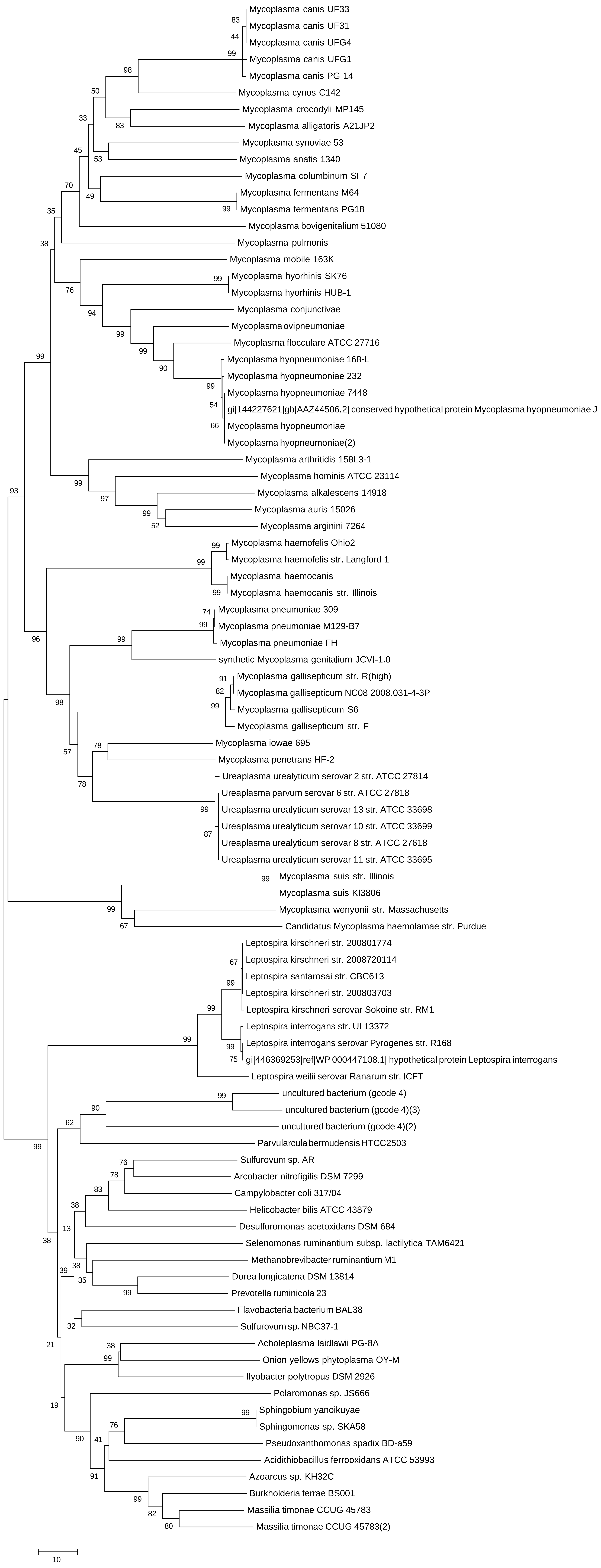
Mycoplasma canis UF33
Mycoplasma canis UF31
Mycoplasma canis UFG4
Mycoplasma canis UFG1
Mycoplasma canis PG 14
Mycoplasma cynos C142
Mycoplasma crocodyli MP145
Mycoplasma alligatoris A21JP2
Mycoplasma synoviae 53
Mycoplasma anatis 1340
Mycoplasma columbinum SF7
Mycoplasma fermentans M64
Mycoplasma fermentans PG18
Mycoplasma bovigenitalium 51080
Mycoplasma pulmonis
Mycoplasma mobile 163K
Mycoplasma hyorhinis SK76
Mycoplasma hyorhinis HUB-1
Mycoplasma conjunctivae
Mycoplasma ovipneumoniae
Mycoplasma flocculare ATCC 27716
Mycoplasma hyopneumoniae 168-L
Mycoplasma hyopneumoniae 232
Mycoplasma hyopneumoniae 7448
gi|144227621|gb|AAZ44506.2| conserved hypothetical protein Mycoplasma hyopneumoniae J
Mycoplasma hyopneumoniae
Mycoplasma hyopneumoniae(2)
Mycoplasma arthritidis 158L3-1
Mycoplasma hominis ATCC 23114
Mycoplasma alkalescens 14918
Mycoplasma auris 15026
Mycoplasma arginini 7264
Mycoplasma haemofelis Ohio2
Mycoplasma haemofelis str. Langford 1
Mycoplasma haemocanis
Mycoplasma haemocanis str. Illinois
Mycoplasma pneumoniae 309
Mycoplasma pneumoniae M129-B7
Mycoplasma pneumoniae FH
synthetic Mycoplasma genitalium JCVI-1.0
Mycoplasma gallisepticum str. R(high)
Mycoplasma gallisepticum NC08 2008.031-4-3P
Mycoplasma gallisepticum S6
Mycoplasma gallisepticum str. F
Mycoplasma iowae 695
Mycoplasma penetrans HF-2
Ureaplasma urealyticum serovar 2 str. ATCC 27814
Ureaplasma parvum serovar 6 str. ATCC 27818
Ureaplasma urealyticum serovar 13 str. ATCC 33698
Ureaplasma urealyticum serovar 10 str. ATCC 33699
Ureaplasma urealyticum serovar 8 str. ATCC 27618
Ureaplasma urealyticum serovar 11 str. ATCC 33695
Mycoplasma suis str. Illinois
Mycoplasma suis KI3806
Mycoplasma wenyonii str. Massachusetts
Candidatus Mycoplasma haemolamae str. Purdue
Leptospira kirschneri str. 200801774
Leptospira kirschneri str. 2008720114
Leptospira santarosai str. CBC613
Leptospira kirschneri str. 200803703
Leptospira kirschneri serovar Sokoine str. RM1
Leptospira interrogans str. UI 13372
Leptospira interrogans serovar Pyrogenes str. R168
gi|446369253|ref|WP 004447108.1| hypothetical protein Leptospira interrogans
Leptospira weilii serovar Ranarum str. ICFT
uncultured bacterium (gcode 4)
uncultured bacterium (gcode 4)(3)
uncultured bacterium (gcode 4)(2)
Parvularcula bermudensis HTCC2503
Sulfurovum sp. AR
Arcobacter nitrofigilis DSM 7299
Campylobacter coli 317/04
Helicobacter bilis ATCC 43879
Desulfuromonas acetoxidans DSM 684
Selenomonas ruminantium subsp. lactilytica TAM6421
Methanobrevibacter ruminantium M1
Dorea longicatena DSM 13814
Prevotella ruminicola 23
Flavobacteria bacterium BAL38
Sulfurovum sp. NBC37-1
Acholeplasma laidlawii PG-8A
Onion yellows phytoplasma OY-M
Ilyobacter polytropus DSM 2926
Polaromonas sp. JS666
Sphingobium yanoikuyae
Sphingomonas sp. SKA58
Pseudoxanthomonas spadix BD-a59
Acidithiobacillus ferrooxidans ATCC 53993
Azoarcus sp. KH32C
Burkholderia terrae BS001
Massilia timonae CCUG 45783
Massilia timonae CCUG 45783(2)
10

Figure S9. Phylogenetic tree of the horizontal gene transfer analysis. This tree is the phylogenetic reconstruction of the 100 first hits retrieved from the BLAST search using MHF_0804 protein sequence (hypothetical protein, *M. haemofelis* Langford 1) (Table 3). Sequences were aligned using MUSCLE [21] and the tree constructed using neighbor-joining [28] with 1,000 bootstrap replicates in MEGA 5 [22]. This tree was manually compared to a 16S rRNA gene phylogenetic tree to infer HGT as previously described [30].
