# Supplementary material for: Comparative Genomics and Phylogenomics of Hemotrophic Mycoplasmas
Source: PLoS One. 2014 Mar 18;9(3):e91445. doi: 10.1371/journal.pone.0091445 (PMC3958358; doi:10.1371/journal.pone.0091445)
Supplement: Supplementary Material S1 — Horizontal gene transfer analysis. (PDF) [file pone.0091445.s010.pdf]

A total of 30,546 proteins from *Mollicutes* (Table S1) were analyzed using OrthoMCL, of which 6 proteins were less than 30 amino acids long and were excluded. From the remaining 30,540 proteins, 19,158 formed 2,789 COGs. A total of 1,017 COGs contained at least one hemoplasma species (COGh) and were selected for further analysis (Figure Supplementary Material 1.1). Among these, 104 clusters contained proteins of all 35 proteomes (core proteome including *Bacillus subtillis*) and were therefore not included in the HGT analyses. Of the remaining 913 clusters, 645 (70.65%) were composed of *M. haemofelis* and *M. haemocanis* orthologs only. The presence or absence of proteins (genes) in each of the 913 clusters was mapped onto the species tree (Figure S3) and gene gain events were annotated. At least one gene gain event related to hemoplasmas was observed in 801 clusters (645 *M. haemofelis* & *M. haemocanis* clusters + 156 other clusters).

A hemoplasma representative of each of the 801 clusters was selected and subjected to BLASTp analysis; the 100 first hits were then used for phylogenetic reconstruction and HGT detection. Among the 801 clusters with at least one gene gain event, 702 (87.64%) had no significant BLASTp hits outside of the hemoplasma group. From the remaining 99 clusters, phylogenetic reconstruction was successful for 90 clusters, but weakly supported bootstrap values were observed in 29 (out of the 90; 32.22%) cases. Finally, six (6 out of 64; 9.37%) COGs/genes showed phylogenetic support for putative HGT (Table 3).

From the 5,670 proteins of the 6 hemoplasma species, 2,853 proteins did not form ortholog groups. These proteins were analyzed in the same manner as described above. Only 22 (0.77%) proteins showed significant BLASTp hits. Phylogenetic reconstruction was successful in 21 cases, but low bootstrap values were observed in 11 cases and no cases of HGT were detected.

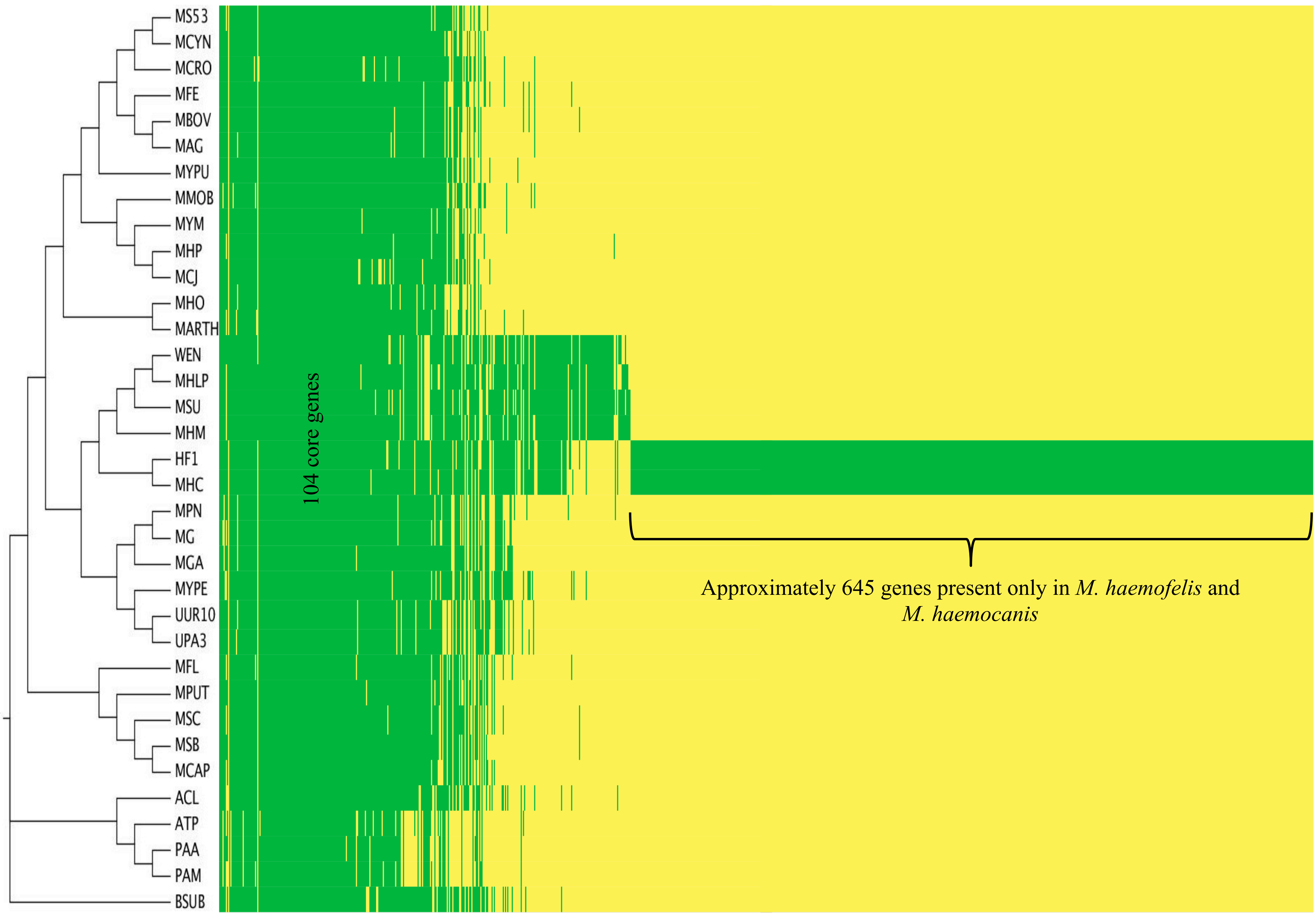

Figure Supplementary Material 1.1. Presence or absence of genes (n=1,017 COGhs) mapped onto the 16S rRNA phylogenetic tree. Green means "gene is present' and yellow means "gene is absent". Note 104 core genes (present in all Mollicutes and *Bacillus subtillis*, BSUB) at the center and orthologous genes of present only in *M. haemofelis* (HF1) and *M. haemocanis* (MHC).
