# Supplementary material for: Comparative Genomics and Phylogenomics of Hemotrophic Mycoplasmas
Source: PLoS One. 2014 Mar 18;9(3):e91445. doi: 10.1371/journal.pone.0091445 (PMC3958358; doi:10.1371/journal.pone.0091445)
Supplement: Table S1 — Bacterial organisms used in the horizontal gene transfer (HGT) and phylogeny studies. (DOCX) [file pone.0091445.s011.docx]

**Table S1**. Bacterial organisms used in the horizontal gene transfer (HGT) and phylogeny studies.

|  | **BioProject** | **Organism** | **Analysis** |
| --- | --- | --- | --- |
| **1** | PRJNA82367 | *M. haemocanis* str. Illinois | HGT & Phylogeny |
| **2** | PRJNA61897 | *M. suis* str. Illinois | HGT & Phylogeny |
| **3** | PRJNA162029 | *M. haemofelis* strain Ohio2 | HGT & Phylogeny |
| **4** | PRJNA168067 | *M. wenyonii* str. Massachusetts | HGT & Phylogeny |
| **5** | PRJNA68151 | ‘*Candidatus* M. haemolamae’ str. Purdue | HGT & Phylogeny |
| **6** | PRJNA76933 | ‘*Candidatus* M. haemominutum’ str. Birmingham 1 | HGT & Phylogeny |
| **7** | PRJNA58887 | *U. parvum* serovar 3 str. ATCC 27815 | HGT & Phylogeny |
| **8** | PRJNA59011 | *U. urealyticum* serovar 10 str. ATCC 33699 | HGT & Phylogeny |
| **9** | PRJNA57729 | *M. penetrans* HF-2 | HGT & Phylogeny |
| **10** | PRJNA57709 | *M. pneumoniae* M129 | HGT & Phylogeny |
| **11** | PRJNA57707 | *M. genitalium* G37 | HGT & Phylogeny |
| **12** | PRJNA161999 | *M. gallisepticum* str. R(high) | HGT & Phylogeny |
| **13** | PRJNA184824 | *M. cynos* C142 | HGT & Phylogeny |
| **14** | PRJEA53147 | *M. hominis* ATCC 23114 | HGT & Phylogeny |
| **15** | PRJNA58005 | *M. arthritidis* 158L3-1 | HGT & Phylogeny |
| **16** | PRJNA58077 | *M. mobile* 163K | HGT & Phylogeny |
| **17** | PRJNA61569 | *M. pulmonis* UAB CTIP | HGT & Phylogeny |
| **18** | PRJNA87003 | *M. hyorhinis* GDL-1 | HGT & Phylogeny |
| **19** | PRJNA58205 | *M. hyopneumoniae* 232 | HGT & Phylogeny |
| **20** | PRJNA59325 | *M. conjunctivae* HRC/581 | HGT & Phylogeny |
| **21** | PRJNA58061 | *M. synoviae* 53 | HGT & Phylogeny |
| **22** | PRJNA47087 | *M. crocodyli* MP145 | HGT & Phylogeny |
| **23** | PRJNA197154 | *M. fermentans* PG18 | HGT & Phylogeny |
| **24** | PRJNA60859 | *M. bovis* PG45 | HGT & Phylogeny |
| **25** | PRJNA61619 | *M. agalactiae* PG2 | HGT & Phylogeny |
| **26** | PRJNA58525 | *M. capricolum* subsp. *capricolum* ATCC 27343 | HGT & Phylogeny |
| **27** | PRJNA60849 | *M. leachii* PG50 | HGT & Phylogeny |
| **28** | PRJNA58031 | *M. mycoides* subsp. *mycoides* SC str. PG1 | HGT & Phylogeny |
| **29** | PRJNA72481 | *M. putrefaciens* KS1 | HGT & Phylogeny |
| **30** | PRJNA58055 | *Mesoplasma florum* L1 | HGT & Phylogeny |
| **31** | PRJNA61641 | ‘*Candidatus* Phytoplasma australiense’ | HGT & Phylogeny |
| **32** | PRJNA58015 | Onion yellows phytoplasma OY-M | HGT & Phylogeny |
| **33** | PRJNA59087 | ‘*Candidatus* P. mali’ | HGT & Phylogeny |
| **34** | PRJNA58901 | *Acholeplasma laidlawii* PG-8A | HGT & Phylogeny |
| **35** | PRJNA55263 | *Bacillus subtillis* subsp. *subtillis* str. 168 | HGT & Phylogeny |
| **36** | PRJNA63665 | *M. suis* strain KI3806 | Phylogeny |
| **37** | PRJNA62461 | *M. haemofelis* strain Langford1 | Phylogeny |
| **38** | PRJNA74019 | *M. iowae* 695 | Phylogeny |
| **39** | PRJNA85495 | *M. pneumoniae* 309 | Phylogeny |
| **40** | PRJNA57993 | *M. gallisepticum* str. R(low) | Phylogeny |
| **41** | PRJNA79051 | *M. ovipneumoniae* SC01 | Phylogeny |
| **42** | PRJNA58039 | *M. hyopneumoniae* 7448 | Phylogeny |
| **43** | PRJNA58059 | *M. hyopneumoniae* J | Phylogeny |
| **44** | PRJNA71183 | *M. anatis* 1340 | Phylogeny |
| **45** | PRJNA53543 | *M. fermentans* JER | Phylogeny |
| **46** | PRJNA46679 | *M. agalactiae* 5632 | Phylogeny |
| **47** | PRJNA27713 | *M. mycoides* subsp. *mycoides* SC str. Gladysdale | Phylogeny |
| **48** | PRJEA162031 | *M. leachii* 99/014/6 | Phylogeny |
| **49** | PRJNA66189 | *M. mycoides* subsp. *capri* LC str. 95010 | Phylogeny |
| **50** | PRJNA78319 | *Spiroplasma melliferum* KC3 | Phylogeny |
| **51** | PRJNA58297 | Aster yellows witches’-broom phytoplasma AYWB | Phylogeny |
| **52** | PRJNA62099 | *M. fermentans* M64 | Phylogeny |
| **53** | PRJNA68691 | *M. bovis* Hubei-1 | Phylogeny |
| **54** | PRJNA46947 | *M. alligatoris* A21JP2 | Phylogeny |
