# Supplementary material for: Comparative Genomics and Phylogenomics of Hemotrophic Mycoplasmas
Source: PLoS One. 2014 Mar 18;9(3):e91445. doi: 10.1371/journal.pone.0091445 (PMC3958358; doi:10.1371/journal.pone.0091445)
Supplement: Table S2 — Conserved bacterial genes used as control for the positive selection analysis. (DOCX) [file pone.0091445.s012.docx]

**Table S2**. Conserved bacterial genes used as control for the positive selection analysis.

| **ID** | **Annotation** | **Gene ID*** | **Number of negatively selected codon-sites** |
| --- | --- | --- | --- |
| 1 | Enolase | MSU_0703 | 242 |
| 2 | Glyceraldehyde 3-phosphate dehydrogenase | MSU_0835 | 177 |
| 3 | DNA gyrase subunit B | MSU_0030 | 263 |
| 4 | Preprotein translocase subunit SecY | MSU_0438 | 232 |
| 5 | Signal recognition particle-docking protein FtsY | MSU_0832 | 109 |
| 6 | 30S ribosomal protein S3 | MSU_0426 | 105 |
| 7 | Translation initiation factor IF-1 | MSU_0443 | 27 |
| 8 | 50S ribosomal protein L11 | MSU_0295 | 57 |
| 9 | Elongation factor P | MSU_0724 | 68 |
| 10 | Thymidine kinase | MSU_0066 | 79 |
| 11 | Oligoendopeptidase F | MSU_0828 | 209 |
| 12 | Ribonuclease P | MSU_0884 | 34 |
| 13 | Glucose 6-phosphate isomerase | MSU_0013 | 169 |
| 14 | Fructose 1,6-biphosphate aldolase | MSU_0475 | 153 |
| 15 | Pyruvate kinase | MSU_0701 | 199 |
| 16 | Phosphoglycerate kinase | MSU_0834 | 181 |
| 17 | Phosphoglyceromutase | MSU_0072 | 186 |
| 18 | L-lactate dehydrogenase | MSU_0299 | 132 |
| 19 | Triosephosphate isomerase | MSU_0071 | 89 |
| 20 | 6-phosphofructokinase | MSU_0702 | 132 |
| 21 | CTP synthase | MSU_0362 | 203 |
| 22 | Phosphocarrier protein HPr | MSU_0457 | 32 |
| 23 | Recombinase A | MSU_0830 | 146 |
| 24 | Uridylate kinase | MSU_0503 | 87 |
| 25 | 30S ribosomal protein S13 | MSU_0447 | 78 |
| 26 | 30S ribosomal protein S10 | MSU_0418 | 46 |
| 27 | 30S ribosomal protein S19 | MSU_0424 | 44 |
| 28 | Inorganic pyrophosphatase | MSU_0783 | 71 |
| 29 | Cardiolipin synthase | MSU_0014 | 170 |
| 30 | DNA gyrase subunit A | MSU_0031 | 290 |

* *Mycoplasma suis* strain Illinois was used as example for gene ID. No positively selected sites were found in any of these sequences.
