# Supplementary material for: Comparative Genomics and Phylogenomics of Hemotrophic Mycoplasmas
Source: PLoS One. 2014 Mar 18;9(3):e91445. doi: 10.1371/journal.pone.0091445 (PMC3958358; doi:10.1371/journal.pone.0091445)
Supplement: Table S3 — Cluster of orthologous groups (COG)/proteins used in the phylogenetic analysis. (DOCX) [file pone.0091445.s013.docx]

**Table S3**. Cluster of orthologous groups (COG)/proteins used in the phylogenetic analysis.

| **ID** | **COG** | **Annotation** | **Av. size** |
| --- | --- | --- | --- |
| **1** | COG0012 | Predicted GTPase, probable translation factor | 371 |
| **2** | COG0016 | Phenylalanine-tRNA synthetase alpha subunit | 325 |
| **3** | COG0048 | Ribosomal protein S12 | 139 |
| **4** | COG0049 | Ribosomal protein S7 | 156 |
| **5** | COG0050 | GTPases, translation elongation factors, factor Tu (EF-Tu) | 396 |
| **6** | COG0080 | Ribosomal protein L11 | 153 |
| **7** | COG0087 | Ribosomal protein L3 | 246 |
| **8** | COG0093 | Ribosomal protein L14 | 122 |
| **9** | COG0096 | Ribosomal protein S8 | 132 |
| **10** | COG0097 | Ribosomal protein L6P/L9E | 181 |
| **11** | COG0098 | Ribosomal protein S5 | 221 |
| **12** | COG0100 | Ribosomal protein S11 | 128 |
| **13** | COG0102 | Ribosomal protein L13 | 146 |
| **14** | COG0103 | Ribosomal protein S9 | 134 |
| **15** | COG0184 | Ribosomal protein S15P/S13E | 85 |
| **16** | COG0188 | TypeIIA topoisomerase (DNA gyrase/topo II, topositomarease IV), A subunit | 867 |
| **17** | COG0197 | Ribosomal protein L16/L10E | 139 |
| **18** | COG0200 | Ribosomal protein L15 | 147 |
| **19** | COG0201 | Preprotein translocase subunit SecY | 476 |
| **20** | COG0202 | DNA-directed RNA polymerase, alpha subunit | 330 |
| **21** | COG0231 | Translation elongation factor P (EF-P) | 185 |
| **22** | COG0256 | Ribosomal protein L18 | 118 |
| **23** | COG0443 | Molecular chaperone DnaK (Hsp70) | 598 |
| **24** | COG0480 | Translation elongation factors (GTPases), factor G (EF-G) | 692 |
| **25** | COG0522 | Ribosomal protein S4 and related proteins | 203 |
| **26** | COG0533 | Metal dependent proteases with possible chaperone activity | 315 |
| **27** | COG0552 | Signal recognition particle GTPase, FtsY | 353 |
| **28** | COG0094 | Ribosomal protein L5 | 182 |
| **29** | COG0099 | Ribosomal protein S13 | 130 |
| **30** | COG0172 | Seryl-tRNA synthetase | 419 |
| **31** | COG0186 | Ribosomal protein S17 | 88 |
| **32** | COG0495 | Leucyl-tRNA synthetase | 782 |
