# Supplementary material for: Comparative Genomics and Phylogenomics of Hemotrophic Mycoplasmas
Source: PLoS One. 2014 Mar 18;9(3):e91445. doi: 10.1371/journal.pone.0091445 (PMC3958358; doi:10.1371/journal.pone.0091445)
Supplement: Table S4 — Number of Clusters of Orthologous Groups (COGs) between hemoplasma species. (DOCX) [file pone.0091445.s014.docx]

**Table S4.** Number of Clusters of Orthologous Groups (COGs) between hemoplasma species.

|  | *M. haemolamae* | *M. haemofelis* Ohio2 | *M. haemofelis* Langford | *M. haemocanis* | *M. suis* Illinois | *M. suis* KI3806 | *M. haemominutum* | *M. wenyonii* |
| --- | --- | --- | --- | --- | --- | --- | --- | --- |
| *M. haemolamae* | - | 262 | 268 | 268 | 305 | 314 | 308 | 291 |
| *M. haemofelis* Ohio2 | 262 | - | 1381 | 935 | 265 | 268 | 232 | 261 |
| *M. haemofelis* Langford | 268 | 1381 | - | 944 | 271 | 274 | 274 | 268 |
| *M. haemocanis* | 268 | 935 | 944 | - | 268 | 271 | 274 | 268 |
| *M. suis* Illinois | 305 | 265 | 271 | 268 | - | 623 | 315 | 289 |
| *M. suis* KI3806 | 314 | 268 | 274 | 271 | 623 | - | 319 | 295 |
| *M. haemominutum* | 308 | 232 | 274 | 274 | 315 | 319 | - | 294 |
| *M. wenyonii* | 291 | 261 | 268 | 268 | 289 | 295 | 294 | - |
