# Supplementary material for: Comparative Genomics and Phylogenomics of Hemotrophic Mycoplasmas
Source: PLoS One. 2014 Mar 18;9(3):e91445. doi: 10.1371/journal.pone.0091445 (PMC3958358; doi:10.1371/journal.pone.0091445)
Supplement: Table S5 — Putative horizontally transferred genes among members of the suis groups and cases in which gene duplication could not be distinguished from HGT. (DOCX) [file pone.0091445.s015.docx]

**Table S5**. Putative horizontally transferred genes among members of the suis groups and cases in which gene duplication could not be distinguished from HGT.

| **Putative horizontally transferred genes of the suis group** | |
| --- | --- |
| **Gene ID*** | **Annotation** |
| MHC_00920 | MgPa-like protein |
| MHF_1614 | 60Kd inner membrane protein |
| MHF_0769 | 30S ribosomal protein S21 |
| MHF_0065 | Hypothetical protein |
| MHF_1171 | aldo/keto reductase family oxidoreductase |
| MHF_1588 | spermidine/putrescine import ATP-binding protein PotA |
| MHF_0045 | MFS transporter |
| MHF_1568 | adenylosuccinate synthetase |
| MHC_05485 | Hypothetical protein |
| MHC_00940 | Hypothetical protein |
| MHF_1174 | aldo/keto reductase family oxidoreductase |
| MHF_1223 | ferrichrome ABC transporter ATP-binding protein |
| MHF_1220 | ferrichrome ABC transporter |
| MHF_1213 | nicotinate-nucleotide adenylyltransferase |
| MHF_0251 | sodium/calcium exchanger protein |
| MHF_0138 | comEC/Rec2-related domain protein |
| MHF_0147 | ATP synthase F1 subunit delta |
| MHF_1608 | Hypothetical protein |
| **Cases in which gene duplication could not be distinguished from HGT** | |
| **Gene ID*** | **Annotation** |
| HF1_00530 | RNA polymerase sigma factor rpoD |
| WEN_02145 | Hypothetical protein |
| MSU_0794 | Hypothetical protein |
| MHF_0247 | Hypothetical protein |
| MHF_0803 | Hypothetical protein |
| WEN_03070 | Hypothetical protein |
| MHC_05515 | adenylosuccinate lyase/hypoxanthine phosphoribosyltransferase |

* One representative gene is listed.
